# A *Helitron* transposon reconstructed from bats reveals a novel mechanism of genome shuffling in eukaryotes

**DOI:** 10.1038/ncomms10716

**Published:** 2016-03-02

**Authors:** Ivana Grabundzija, Simon A. Messing, Jainy Thomas, Rachel L. Cosby, Ilija Bilic, Csaba Miskey, Andreas Gogol-Döring, Vladimir Kapitonov, Tanja Diem, Anna Dalda, Jerzy Jurka, Ellen J. Pritham, Fred Dyda, Zsuzsanna Izsvák, Zoltán Ivics

**Affiliations:** 1 Max Delbrück Center for Molecular Medicine, 13125 Berlin, Germany; 2 Laboratory of Molecular Biology, NIDDK, NIH, Bethesda, Maryland 20892, USA; 3 Department of Human Genetics, University of Utah, Salt Lake City, Utah 84112, USA; 4 Division of Medical Biotechnology, Paul Ehrlich Institute, 63225 Langen, Germany; 5 German Center of Integrative Biodiversity Research (iDiv), 04103 Halle-Jena-Leipzig, Germany; 6 Institute of Computer Science, Martin Luther University Halle–Wittenberg, 06099 Halle, Germany; 7 Genetic Information Research Institute, Los Altos, California 94022, USA

## Abstract

*Helitron* transposons capture and mobilize gene fragments in eukaryotes, but experimental evidence for their transposition is lacking in the absence of an isolated active element. Here we reconstruct *Helraiser*, an ancient element from the bat genome, and use this transposon as an experimental tool to unravel the mechanism of *Helitron* transposition. A hairpin close to the 3′-end of the transposon functions as a transposition terminator. However, the 3′-end can be bypassed by the transposase, resulting in transduction of flanking sequences to new genomic locations. *Helraiser* transposition generates covalently closed circular intermediates, suggestive of a replicative transposition mechanism, which provides a powerful means to disseminate captured transcriptional regulatory signals across the genome. Indeed, we document the generation of novel transcripts by *Helitron* promoter capture both experimentally and by transcriptome analysis in bats. Our results provide mechanistic insight into *Helitron* transposition, and its impact on diversification of gene function by genome shuffling.

## Abstract (web summary)

*Helitron* elements are proposed rolling-circle transposons in eukaryotic genomes, but experimental evidence for their transposition has been lacking. Here, Grabundzija *et al*. reconstruct an active *Helitron* from bats which they name *Helraiser*, and characterize its mechanism of transposition in cell-free reactions and in human cell cultures *in vitro*.

## Graphical abstract

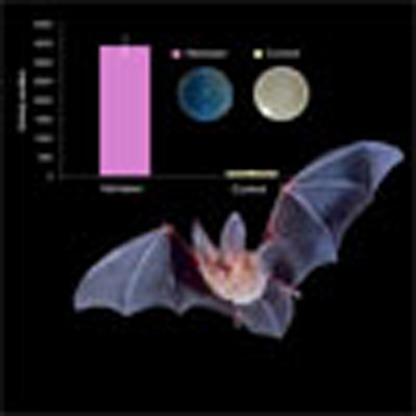

Due to their numbers and mobility, transposable elements are important players in genome evolution. Transposable elements can amplify to high copy numbers despite control by silencing mechanisms. However, accumulation of disabling mutations in their sequences leads to transpositional inactivation and subsequent extinction. Thus, ancient transposable elements can often be discovered and annotated only by bioinformatic means. *Helitrons*, a novel group of DNA transposons widespread throughout eukaryotes, were discovered by *in silico* genome-sequence analysis (reviewed in refs 1, 2).

*Helitron* transposition displays a number of features unusual for DNA transposons, such as lack of target site duplications (reviewed in refs 1, 2). Furthermore, putative *Helitron* transposases do not contain an RNase-H-like catalytic domain^3^, but encode a ‘RepHel' motif made up by a replication initiator (Rep) and a DNA helicase (Hel) domain^1^,^2^,^4^. Rep is a nuclease domain of the HUH superfamily of nucleases involved in catalytic reactions for endonucleolytic cleavage, DNA transfer and ligation^5^,^6^. HUH nucleases cleave exclusively single-stranded DNA (ssDNA), and have a key role in the initiation of ‘rolling-circle replication' of certain bacteriophages such as φX174 (ref. 7), ssDNA viruses and bacterial plasmids (reviewed in ref. 8), as well as in ‘rolling-circle' transposition of IS*91* family bacterial transposons^9^,^10^,^11^.

The key elements of the proposed rolling-circle transposition mechanism^12^ involve two tyrosine (Tyr) residues in the active site of IS*91*'s HUH transposase^9^. Briefly, the model proposes a site-specific nick at the 5′-end of the transposon, with the transposase forming a 5′-phosphotyrosine intermediate. The 3′-OH at the nick serves to initiate DNA synthesis while one transposon DNA strand peels off. The nick generated in the target DNA possibly by the second active site Tyr leads to the resolution of the 5′-phosphotyrosine. Once the entire transposon has been replicated, the transposase catalyses a second strand-transfer event by nicking the 3′-end of the transposon and joining it to the 5′-end of the target site^1^,^8^,^11^. It has been suggested that *Helitrons* are the first eukaryotic rolling-circle transposons^4^, although definite information involving their transposition mechanism remains elusive due to the lack of an active element isolated from any species.

The only *Helitron* transposons found in sequenced mammalian genomes are from vespertilionid bats^13^,^14^,^15^. In contrast to other DNA transposons, the *Helibat* family was active throughout the diversification of vespertilionid bats from 30 to 36 myr ago to as recently as 1.8–6 myr ago^14^. *Helibats* comprise ∼6 % of the little brown bat (*Myotis lucifugus*) genome^14^, where the autonomous *Helibat1* elements and multiple non-autonomous subfamilies including *HelibatN1*, *HelibatN2* and *HelibatN3* have been amplified to >100,000 copies^13^,^14^. The predicted transposase encoded by bat *Helitrons* contains the typical ‘RepHel' motif, the elements are characterized by 5′-TC and CTRR-3′ termini that do not contain inverted repeats but have a short palindromic motif located upstream of the 3′-terminus, and insertions occurred precisely between 5′-A and T-3′ nucleotides at host AT target sites^13^. Although the vast majority of *Helitron* families harbour short palindromic sequences in their 3′-termini^4^,^16^,^17^,^18^,^19^, the role of these sequences in *Helitron* transposition is unclear.

Genomic data suggest that *Helitron* transposition is often associated with the capture and mobilization of host genomic fragments, resulting in the dissemination of genomic regulatory elements^13^,^14^, gene fragment duplications^20^, the generation of chimeric transcripts^14^,^20^ and the creation of putative microRNA-binding sites^14^. This process appears to have been particularly frequent in the maize (*Zea mays*) genome, where some *Helitrons* have been shown to carry exons transduced from as many as 12 genes^21^. These observations imply a significantly higher impact on genomes by *Helitrons* than by other DNA transposons. Although prokaryotic IS*91*-like elements have been implicated in co-mobilization of adjacent bacterial genes, including antibiotic resistance genes, evidence for a transduction mechanism remains circumstantial^22^,^23^. Likewise, although several mechanisms have been proposed to explain *Helitron* gene capture^1^,^2^,^16^,^21^,^24^,^25^,^26^, due to the lack of direct experimental data, both the process and regulation of *Helitron* transposition has remained enigmatic.

Everything that is known to date about *Helitron* biology derives from *in silico* or genetic analysis, because no active *Helitron* transposon has been isolated. Here we reconstruct an active copy of the autonomous *Helibat1* transposon, designated ‘*Helraiser*', and characterize its transposition *in vitro* and in human cells *ex vivo*. We provide experimental insight into the transposition of *Helitrons* by addressing the molecular requirements of transposition, target site selection properties, and gene capture in cell culture and in bats *in vivo*.

## Results

### Structural hallmarks of the resurrected *Helraiser* transposon

**Figure 1:**
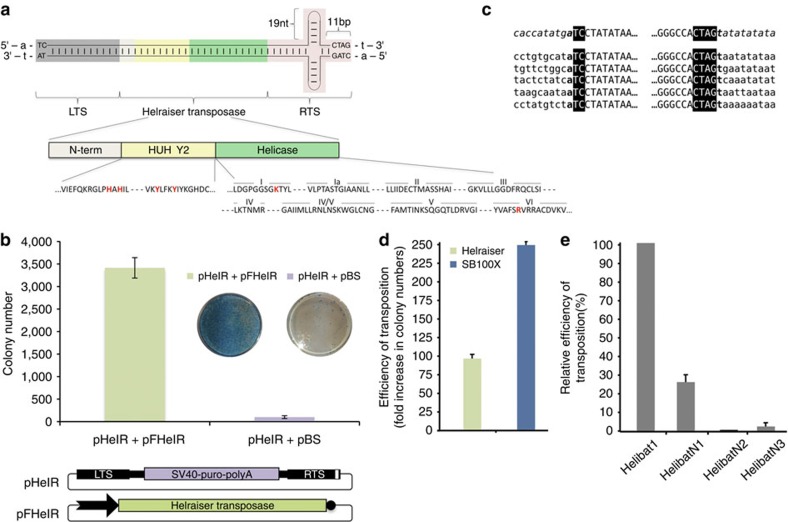
Features of *Helraiser* transposition in human HeLa cells. (**a**) Schematic representation of the *Helraiser* transposon. LTS and RTS terminal sequences are in uppercase, and flanking A and T host target site sequences are in lowercase. White, yellow and green rectangles represent N-terminal, endonuclease and helicase domains of the *Helraiser* transposase, respectively. Conserved amino-acid motifs are shown below. Positions of the residues used for mutagenesis are shown in red. (**b**) *Helraiser* colony-forming efficiency. Shown are tissue culture plates containing stained puro-resistant HeLa cell colonies. *Helraiser* donor (pHelR) and helper (pFHelR) plasmids. White rectangle in RTS: hairpin; black arrow: promoter driving transposase expression, black circle: polyA signal; these annotations are used consistently in all the figures. Data are represented as mean±s.e.m., *n*=7 biological replicates. (**c**) *Helraiser* transposition generates canonical insertions. *Helraiser* LTS- or RTS-to-genome juctions are shown for 10 independent transposon insertions. *Helraiser* sequence is shown in uppercase with the conserved 5′- and 3′-terminal sequences in a black blackground and flanking host genomic sequence is in lowercase. The flanking pHelR plasmid sequence (upper line) is in italic. (**d**) Relative transposition efficiencies of *Helraiser* and *Sleeping Beauty* measured by colony formation in HeLa cells. Data are represented as mean±s.e.m., *n*=3 biological replicates. (**e**) Relative transposition efficiencies of *Helibat1* and the non-autonomous subfamilies *HelibatN1*, *HelibatN2* and *HelibatN3*. Data are represented as mean±s.e.m., *n*=5 biological replicates.

To build a model of an autonomous *Helibat* element, the *M. lucifugus* genome was subjected to bioinformatic analysis (Supplementary Notes). The resulting 5,296-bp *Helraiser* consensus sequence (Supplementary Fig. 1) contains all of the known hallmarks of an autonomous *Helitron* element (reviewed in refs 1, 2). The 1,496-amino-acid (aa)-long coding sequence of the *Helraiser* transposase is flanked by left and right terminal sequences of the transposon, designated LTS and RTS, respectively (Fig. 1a; Supplementary Fig. 1), that terminate with the conserved 5′-TC/CTAG-3′ motifs characteristic of the *Helibat1* family^13^. A 19-bp-long palindromic sequence with the potential to form a hairpin structure is located 11 nucleotides upstream of the RTS end (Fig. 1a; Supplementary Fig. 1).

The *Helraiser* transposase contains a putative, N-terminal nuclear localization signal and a zinc-finger-like motif, followed by a RepHel enzymatic core^4^,^13^. RepHel consists of a ∼300-aa-long Rep nuclease domain, characterized by the conserved HUH motif and two active site Tyr residues, and a ∼600-aa helicase domain containing the eight conserved motifs characteristic of the SF1 superfamily of DNA helicases (Fig. 1a; Supplementary Figs 2A and 3A).

### *Helraiser* transposition in human cells

We synthesized the functional components of the transposon (that is, the transposase as well as the LTS and RTS sequences), and generated a bicomponent transposition system consisting of a puromycin gene (puro)-tagged transposon (designated pHelR) and a transposase-expressing helper plasmid (designated pFHelR; Fig. 1b). As shown in Fig. 1b, transfection of the *Helraiser* system into human HeLa cells generated, on average, ∼3,400 puro-resistant colonies per plate versus ∼100 colonies per plate in the absence of transposase. Thus, the *Helraiser* transposon system appears to contain all of the determinants required for transposition activity in human cells.

Sequence analysis of 10 independent *Helraiser* insertions revealed direct canonical junctions of the transposon LTS 5′-TC motif to an A nucleotide, and of the RTS CTAG-3′ motif to a T nucleotide (Fig. 1c). Thus, *Helitron* transposition into an AT dinucleotide target site was faithfully recapitulated by *Helraiser*.

To evaluate the relative transposition efficiency of *Helraiser*, we directly compared it with a hyperactive variant of *Sleeping Beauty* (SB100X), one of the most active vertebrate cut-and-paste transposons^27^. *Helraiser* demonstrated only about twofold lower colony-forming activity than SB100X in human HeLa cells (Fig. 1d), indicating a relatively high transposition activity even without optimization.

To test the ability of the *Helraiser* transposase to cross-mobilize the non-autonomous transposons *HelibatN1*, *HelibatN2* and *HelibatN3*, their consensus LTS and RTS sequences were synthesized and tagged with neomycin (neo) or puro antibiotic resistance genes, and their transposition activities assayed as described above. *HelibatN1* was the most active (∼28% of the activity of the wild-type *Helraiser* transposon); *HelibatN3* displayed detectable activity (∼2%), whereas *HelibatN2* was apparently inactive under these experimental conditions (Fig. 1e). These data indicate that *Helraiser* represents an ancient *Helibat1* transposase that was probably responsible for mobilizing and propagating at least some of the most abundant non-autonomous *Helitron* subfamilies in the *M. lucifugus* genome.

### Functional analysis of transposase domains

**Figure 2:**
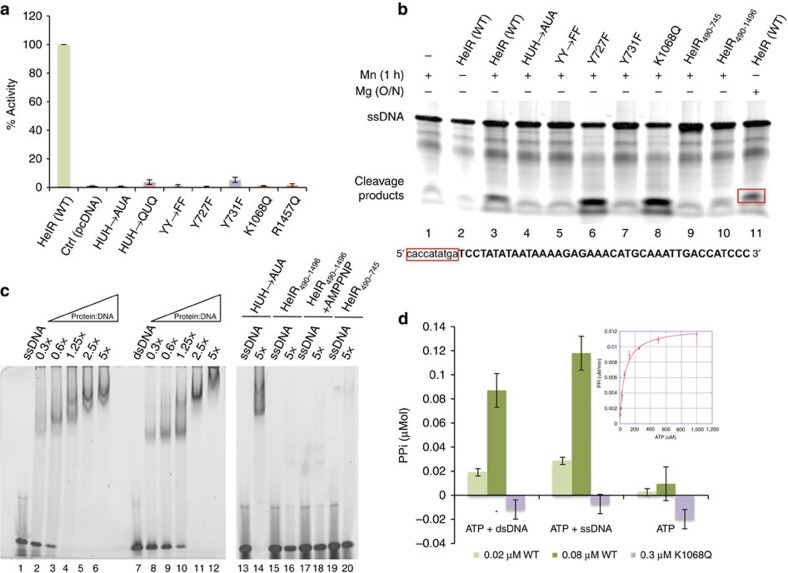
Functional analysis of the HUH nuclease and SF1B helicase domains. (**a**) Transposition by wild-type (WT) *Helraiser* transposase and its mutants in HeLa cells. Data are represented as mean±s.e.m., *n*=3 biological replicates. (**b**) Cleavage of single-stranded DNA oligonucleotides by the *Helraiser* transposase *in vitro*. (**c**) DNA-binding assay with the *Helraiser* transposase and its point mutant and truncated derivatives. 0.3–5 × values represent molar ratios of protein to DNA. (**d**) Colorimetric ATPase assay with the WT and K1068Q mutant transposase protein. PPi, pyrophosphate release. Data are represented as mean ± s.d., *n*=3 biological replicates.

To determine the functional significance of some of the conserved amino acids of the *Helraiser* transposase, we mutated both H593 and H595 and the putative catalytic Y727 and Y731 residues (both individually and together) in the HUH nuclease domain, as well as K1068 of the Walker A motif and the arginine finger R1457 residue located in motif VI of the helicase domain (Fig. 2a). Each of these mutations resulted in loss of transposition activity in HeLa cells (Fig. 2a), suggesting that both nuclease and helicase activities are required for transposition.

*In vitro* assays using purified *Helraiser* transposase demonstrated cleavage of ssDNA (representing 40 bases of the *Helraiser* LTS and RTS plus 10 bases of flanking DNA; Fig. 2b), but not double-stranded DNA (dsDNA; data not shown). Sequencing of the most prominent cleavage products (labelled 1–4, Supplementary Fig. 3B) revealed cleavage between flanking DNA and the *Helraiser* 5′-TC dinucleotide on the top LTS strand (lane 2), between an internal AT dinucleotide on the bottom strand of the LTS (lane 4) and precisely at the transposon end on both strands of the RTS (lanes 6 and 8). These results indicate that the transposon sequence determinants for precise cleavage of the transposon ends are located within the terminal 40 bp on each end.

As expected from an HUH nuclease, cleavage activity required a divalent metal ion (compare lanes 2 and 3, Fig. 2b), and was more efficient with Mn^2+^ than with Mg^2+^ (compare lanes 3 (1 h at 37 °C) and 11 (overnight at 37 °C)). We did not detect ssDNA cleavage on the LTS top strand with either the His–>Ala mutant of the HUH motif (lane 4) or when both Tyr residues were simultaneously mutated (lane 5). We observed a marked difference when the two Tyr residues were individually mutated: mutation of the first Tyr (Y727F) had no effect on cleavage (lane 6), whereas mutation of the second Tyr (Y731F) led to loss of cleavage activity (lane 7). The K1068Q mutation in the helicase domain had no effect on ssDNA cleavage (lane 8). Collectively, these results show that conserved residues of the HUH domain are important for cleavage of ssDNA, and that the two Tyr residues of the active site have distinct roles in *Helitron* transposition.

Limited proteolysis on purified *Helraiser* transposase resulted in three stable fragments corresponding to the N-terminal, the nuclease and the helicase domains (Supplementary Fig. 2B). We used these experimentally determined domain boundaries to design truncated transposases lacking the N-terminal domain and encompassing the nuclease (HelR_490–745_) or nuclease-helicase (HelR_490–1486_) domains. Neither of the purified truncated transposase fragments could cleave DNA (Fig. 2b, lanes 9 and 10), suggesting that the N-terminal domain might be involved in DNA binding. Indeed, as shown in Fig. 2c, although both the wild-type *Helraiser* transposase (lanes 1–12) and the full-length His–>Ala mutant (lanes 13–14 for ssDNA) can bind the oligonucleotides used in the cleavage assays, the truncated versions lacking the N-terminal 489 amino acids did not bind ssDNA (lanes 15–20). These data indicate that the N terminus of the *Helraiser* transposase, containing a predicted zinc-finger-like motif^13^, encodes a DNA-binding domain that is crucial for its ability to bind and cleave ssDNA.

Consistent with helicase activity, the purified transposase hydrolyses ATP with a *K*_m_ of 46±3.3 μM and *k*_cat_ of 6.8±0.11 s^−1^ (inset in Fig. 2d). Importantly, the ATP hydrolysis rate is markedly stimulated by the addition of either dsDNA or ssDNA (Fig. 2d), an effect seen with other SF1 helicases^28^. Mutation of the Walker A motif K1068 abolished ATP hydrolysis (Fig. 2d).

### Role of terminal sequences and 3′-hairpin structure

**Figure 3:**
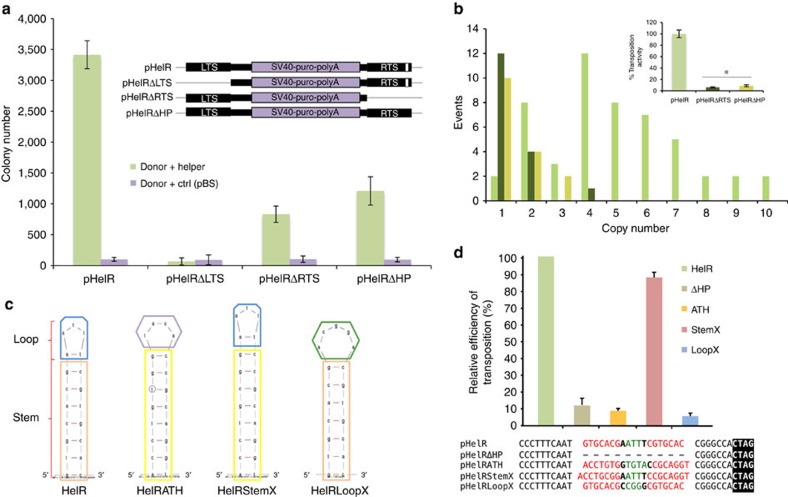
Role of the 3′-terminal sequences and hairpin structure in *Helraiser* transposition. (**a**) Colony-forming efficiencies of the pHelR, pHelRΔLTS, pHelRΔRTS and pHelRΔHP donors. Data are represented as mean±s.e.m., *n*=3 biological replicates. (**b**) Average transposon copy numbers per clone and transposition efficiencies of HelR, HelRΔRTS and HelRΔHP transposons normalized by the average colony numbers (inset). The difference in transposition efficiencies of HelRΔRTS and HelRΔHP transposons is not statistically significant, *P>0.05, unpaired *t*-test. Data are represented as mean±s.e.m., *n*=3 biological replicates. (**c**) M-fold^51^ predicted structures of the HelR, HelRATH, HelRStemX and HelRLoopX hairpins. (**d**) Relative colony-forming activities of hairpin mutants. Data are represented as mean±s.e.m., *n*=3 biological replicates.

To examine the importance of *Helraiser's* terminal sequences on transposition, we created mutants of the transposon vector, pHelRΔLTS and pHelRΔRTS, by deleting either the LTS or the RTS sequences. The presence of the LTS was essential as its deletion abolished *Helraiser* transposition (Fig. 3a). Surprisingly, the presence of the RTS was not essential, although its removal resulted in a decrease of colony-forming activity to ∼24% of the intact transposon (Fig. 3a).

To investigate the role of the RTS further, we created a transposon vector, pHelRΔHP, where the 19-bp palindromic sequence predicted to form a hairpin (‘HP') structure was deleted. As shown in Fig. 3a, pHelRΔHP yielded ∼35% of the transposon colony-forming activity of the intact transposon. Notably, this is comparable to the number of colonies generated with pHelRΔRTS, in which the entire RTS was deleted. Sequence analysis of transposon insertion sites from 51 HeLa clones obtained with the wild-type *Helraiser* transposon indicated an average copy number of 4, with a range between 1 and 10 transposon insertions per clone (Fig. 3b). The same analysis of 16 clones generated with pHelRΔHP, and 15 generated with pHelRΔRTS revealed that both mutant transposons generated, on average, a single insertion per clone (Fig. 3b). Hence, the corrected transposition efficiency of the HelRΔHP and HelRΔRTS transposon mutants are 8.8% and 6% of the transposition efficiency obtained with the wild-type transposon, respectively (inset, Fig. 3b).

To investigate the role of *Helraiser's* RTS hairpin in more depth, we generated three modified transposon donor vectors (pHelRATH, pHelRStemX and pHelRLoopX), in which the hairpin sequence was mutated in different ways (Fig. 3c). In pHelRATH, the *Helraiser* hairpin sequence was replaced with that of the *Helitron1* transposon family in *Arabidopsis thaliana*^4^. pHelRStemX retained the *Helraiser* hairpin loop, whereas the stem sequence was exchanged with that of the *A. thaliana* hairpin. In pHelRLoopX, the stem sequence of the *Helraiser* hairpin was retained but the ATT nucleotides in the loop were replaced with CGG, and the *Helraiser* A–T base pair at the base of the loop was changed to A–A.

Both pHelRATH and pHelRLoopX showed transposition activites similar to pHelRΔHP where the complete palindrome was deleted (Fig. 3d). In contrast, pHelRStemX demonstrated ∼90% of the wild-type transposition activity. These results suggested that, even though the RTS palindrome is not absolutely required for *Helraiser* transposition, it likely plays a role in transposition regulation.

### *Helitron* transposition generates transposon circles

**Figure 4:**
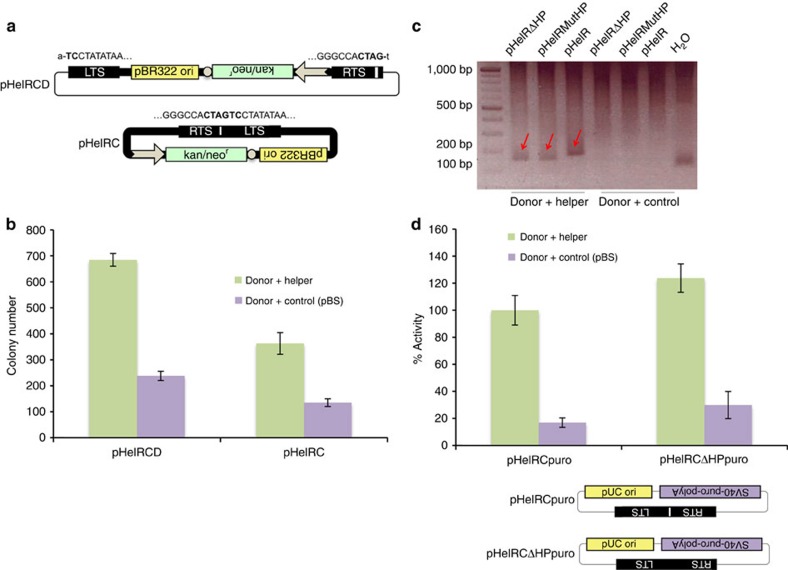
*Helitron* circles. (**a**) *Helitron* circle donor plasmid (pHelRCD) and *Helraiser* transposase-generated *Helitron* circle (pHelRC). White arrow, Kan/SV40 promoter; white circle, polyA signal. (**b**) Transposition of *Helitron* circles. Data are represented as mean±s.e.m., *n*=3 biological replicates. (**c**) PCR detection of *Helitron* circles generated with HelR, HelRMutHP and HelRΔHP transposons. HelRMutHP, transposon with a deletion of the last 9 nt of the palindrome; H_2_O, no template control. (**d**) Relative transposition efficiencies of pHelRCpuro and pHelRCΔHPpuro. Data are represented as mean±s.e.m., *n*=4 biological replicates.

During *Helraiser* insertion site analysis using inverse PCR, we often observed prominent PCR products containing precise head-to-tail junctions of the *Helraiser* transposon ends (the 5′-TC dinucleotide of the LTS is directly and precisely joined to the CTAG-3′ tetranucleotide of the RTS) (Fig. 4a). These data suggested the formation of circular intermediates in *Helraiser* transposition.

To confirm that transposon circles were generated during transposition, we constructed a plasmid-rescue *Helraiser* donor vector, pHelRCD (‘CD': circle donor), in which the transposon LTS and RTS sequences flanked a plasmid replication origin and a kan/neo selection cassette (Fig. 4a). After co-transfection of HeLa cells with pHelRCD and transposase helper plasmids, low-molecular-weight DNA was isolated and electroporated into *E. coli* cells that were subjected to kan selection. One of the 50 *E. coli* colonies contained a *Helraiser*-derived *Helitron* circle (designated ‘pHelRC') consisting of the complete transposon sequence and a perfect head-to-tail-junction of the *Helraiser* LTS and RTS (Fig. 4a). Double-stranded *Helitron* circles are transpositionally active; transposition of pHelRC generated, on average ∼360 colonies per plate, which constitutes ∼51% of the colony-forming efficiency of the plasmid-based pHelRCD *Helitron* circle donor vector (Fig. 4b).

The palindrome in the *Helraiser* RTS is not required for *Helitron* circle formation, because the pHelRΔHP and pHelRMutHP vectors, where the palindrome has been completely or partially deleted, were proficient in generating circles in the presence of *Helraiser* transposase (Fig. 4c). Interestingly, deletion of the palindrome did not have the same detrimental effect on transposition of *Helitron* circles as with plasmid donors, as evidenced by similar colony numbers obtained with pHelRCpuro and pHelRCΔHPpuro in the presence of transposase (Fig. 4d). This result suggests that in the context of transposon circles with joined ends only one nick in the donor DNA has to be made, and thus there is no need to signal the 3′-end of the transposon. In sum, the results indicate the generation of transposon circles as intermediates of *Helraiser* transposition.

### Genome-wide analysis of *Helraiser* insertions

**Figure 5:**
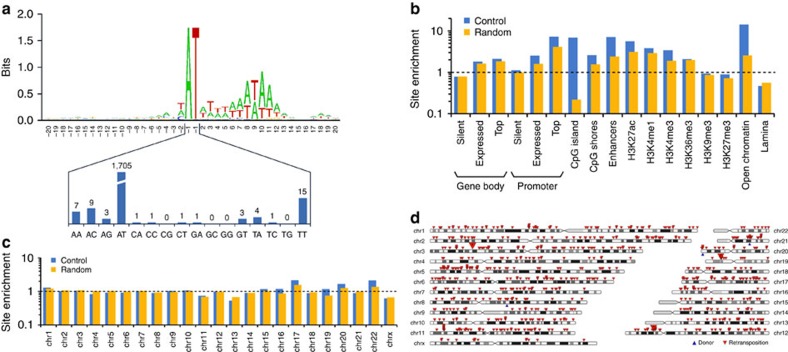
Genome-wide analysis of *de novo Helraiser* insertions in the human genome. (**a**) The sequence logo was created with WebLogo (http://weblogo.berkeley.edu). Transposon integrations are between the positions −1 and 1. The lower panel shows the distribution of dinucleotides at the integration sites. (**b**) Fold enrichment of relative integration frequencies compared with random genomic sites (yellow) and control sites imitating the base composition characteristics of *Helraiser* integration sites (blue). Top genes are the 500 genes with highest expression level. Integration frequencies into promoter regions of silent genes and H3K9me3 regions were not significantly different from controls; all other differences were statistically significant (Fisher's exact test *P* value ≤0.05). (**c**) Fold enrichment of relative integration frequencies per chromosome compared with random genomic sites (yellow) and control sites imitating the base composition characteristics of *Helraiser* integration sites (blue). (**d**) Chromosomal distribution of 701 *Helraiser* retransposition events (red arrows). Blue arrows represent the positions of the original chromosomal donor sites.

Although patterns of *Helitron* insertions have been extensively analysed in the genomes of many eukaryotic species^2^,^13^,^14^,^16^,^17^,^20^,^21^,^29^,^30^,^31^, these patterns are shaped at least in part by natural selection and genetic drift at the level of the host species. By contrast, *de novo* transposition events recovered in cultured cells are subject to hardly any selection or drift (except some possible effects of antibiotic selection), and thus more directly reflect the transposon's integration preferences. To characterize *de novo Helraiser* transposition events in the human genome, we generated, mapped and bioinformatically annotated 1,751 *Helraiser* insertions recovered in HeLa cells. Sequence logo analysis of the targeted sites confirmed AT target dinucleotides as highly preferred sites for integration (Fig. 5a), as previously observed for endogenous *Helitron* transposons in bats and other eukaryotic genomes^1^,^2^,^4^,^13^. However, targeting of AT dinucleotides for insertions was not absolute: 46 insertions occurred into other sequences, with TT, AC and AA being the most prominent alternative dinucleotides (Fig. 5a). In addition to the central AT dinucleotide, we observe a strong preference for an AT-rich DNA sequence within ∼20 bp around the actual integration site; this preference is the most pronounced towards sequences flanking the 3′-end of the integrated transposon (Fig. 5a).

We next analysed relative frequencies of *Helraiser* insertions into different genomic features against computer-generated control datasets of genomic sites that were either picked randomly or modelled by taking into account the base composition observed at transposon insertions (Supplementary Notes). Figure 5b shows a significant, 2.5-fold and 1.8-fold enrichment of *Helraiser* integrations compared with control sites into promoter regions (that is, between 5-kb upstream and 2-kb downstream of transcriptional start sites (TSSs)) and gene bodies (transcription units without their promoter regions), respectively, as defined by the GENCODE catalogue^32^. For both, transcriptional activity appears to positively correlate with integration events because highly expressed genes in HeLa cells are more frequently targeted by *Helraiser* insertions, as evidenced by a 7.3-fold enrichment in promoters and a 2.1-fold enrichment in bodies of the 500 most highly expressed genes (Fig. 5b). In addition, *Helraiser* shows a strong, 6.9-fold enrichment for integration into CpG islands over base composition-corrected control sites, CpG shores (2.6-fold enrichment over control sites in 5-kb windows flanking CpG islands), enhancer regions (derived from CAGE (cap analysis of gene expression) peaks^33^, 7.1-fold enrichment), chromosomal regions enriched for the histone modifications H3K27ac (enhancer, 5.6-fold), H3K4me1 (enhancer, 3.8-fold), H3K4me3 (active promoter, 3.4-fold), H3K36me3 (transcribed gene body, 2.1-fold) and open chromatin regions as defined by DNaseI footprinting, FAIRE (formaldehyde-assisted isolation of regulatory elements) and chromatin immunoprecipitation sequencing experiments (regions taken from the UCSC (University of California, Santa Cruz) Open Chrom Synth track, 14.2-fold). On the other hand, we detected a clear lack of preference for transposition into chromosomal regions characterized by the heterochromatin marks H3K9me3 or H3K27me3 and a significant, 2.2-fold underrepresentation of insertions into lamina-associated domains^34^ (Fig. 5b). Finally, there was no correlation between transposon insertion site enrichment and gene density (Fig. 5c). We have analysed the genomic distribution of inactive *Helitrons* in the bat genome, and found that, in contrast to *de novo* transposition events, endogenous elements are underrepresented near TSSs (where the impact of an insertion on gene expression is expected to be high; Supplementary Table 1), suggesting biological selection against most of these events.

To test whether *Helraiser* exhibits preference for mobilization into *cis*-linked loci when transposition is initiated from genomic donor sites (often seen with many ‘cut-and-paste' transposons and termed ‘local hopping'^35^,^36^,^37^,^38^), we employed a transposon donor cell line containing three identified chromosomal *Helraiser* donor sites, and retransfected these cells with a transposase helper plasmid to drive secondary transposition events to new chromosomal sites. Analysis of 701 retransposition insertion sites revealed no clustering of the new transposon insertions around the original donor sites (Fig. 5d).

### Gene capture by *Helraiser*

Our results presented in Fig. 3a demonstrated that some transposition could take place even if the entire RTS was missing. This raises the question of what sequence determinants define the 3′-end of the mobilized DNA segment.

**Figure 6:**
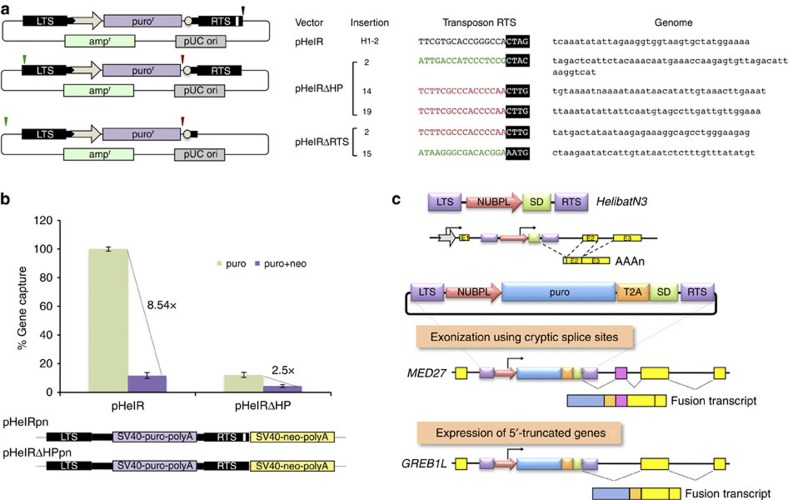
Mechanism of *Helraiser* gene capture. (**a**) Identification of novel 3′-terminal sequences generated by pHelRΔRTS and pHelRΔHP transposition. Relative positions of canonical and *de novo* 3′-ends generated in pHelR, pHelRΔHP and pHelRΔRTS transposition are indicated by black (canonical RTS), red (truncations) and green (read-through events) arrows. Sequences representing new transposon 3′-terminus-to-genome junctions are shown on the right. (**b**) Gene capture efficiency of HelR and HelRΔHP transposons as measured by transduction of a neomycin resistance cassette. Data are represented as mean±s.e.m., *n*=3 biological replicates. (**c**) *De novo* formation of novel transcripts by *HelibatN3* transposition. On the top, the *HelibatN3* transposon containing a fragment of the *NUBPL* gene containing the promoter and a small piece of the coding region followed by a splice donor (SD) between the left and right terminal sequences (LTS and RTS) of the transposon is depicted. In the middle, a *HelibatN3* transposon tagged with a puromycin resistance selectable marker is shown. The T2A self-cleaving peptide sequence allows processing of the primary fusion protein to allow more reliable puro expression. On the bottom, two examples show exonization of noncoding RNA and truncation of mRNAs by imposed splicing. *MED27*, mediator complex subunit 27 gene; *GREB1L*, growth regulation by oestrogen in breast cancer-like gene.

DNA sequencing of insertion sites generated by pHelR, pHelRΔHP and pHelRΔRTS revealed canonical junctions of the LTS 5′-TC sequence to A nucleotides at genomic target sites for all three transposons, indicating that these integrants were indeed *Helraiser* transposase-mediated products. Sequence analysis of the RTS–genome junction revealed the canonical CTAG-3′ sequence flanked by a T nucleotide for pHelR (Fig. 6a; insertion ‘H1-2'). In contrast, some insertions generated by the pHelRΔHP and pHelRΔRTS vectors ended with a CTTG-3′ tetranucleotide (also seen with maize *Helitrons*^21^) inserted immediately adjacent to a T nucleotide at three different genomic target sites (Fig. 6a; shown in red). These transposon insertions represented truncation of the original transposon sequence, since the novel transposon end was situated internally, 6-bp downstream from the start of the SV40 poly A sequence. In addition, two insertions generated by HelRΔHP and HelRΔRTS ended with CTAC-3′ and AATG-3′, respectively (Fig. 6a; shown in green). These events could be considered 3′-transduction events, in which a unique, external sequence representing an alternative transposon RTS has been utilized for transposition. In both cases, the last two nucleotides in the transposon RTS overlapped with the first two nucleotides at the genomic HeLa target site (also seen with one-ended transposition of the IS*91* element^11^), making precise identification of the actual RTSs impossible. None of the five sequences representing the novel RTSs contained an identifiable palindrome within the last 30 bp (data not shown), in line with previous observations^29^.

To further investigate the frequency and extent of 3′-transduction events generated during *Helraiser* transposition, we introduced an SV40-neo-polyA selection cassette immediately downstream of the transposon RTS into the pHelR and pHelRΔHP vectors (renamed ‘pHelRpn' and ‘pHelRΔHPpn' for puro and neo, respectively; Fig. 6b). In this way, read-through events that capture the entire neo cassette can be quantified. As shown in Fig. 6b, the intact *Helraiser* transposon is likely to capture flanking DNA sequence in ∼11.7% of the transposition events. In contrast, although the overall frequency of transposition is lower, at least 36% of the transposition events generated with pHelRΔHPpn resulted in the transfer of the entire 1.6-kb neo cassette downstream of the transposon. This experimental set-up probably gave an underestimate of the frequency of 3′-transduction as it required the capture of the entire 1.6-kb neo cassette.

### Diversification of *Helitron* 3′-ends in *Myotis* genomes

The above experiments demonstrated that premature truncation and read-through events generated through palindrome or RTS deletion leads to the generation of novel 3′-ends. To investigate whether such events have also occurred *in vivo*, we analysed 395 copies of the recently active *HelibatN541*, *HelibatN542* and *HelibatN580* subfamilies (26, 339 and 30 copies, respectively), and found 39 exemplars that have *de novo* 3′-ends (>20% diverged over the last 30 bp of the consensus; Supplementary Table 2). These exemplars were likely generated by (1) insertion adjacent to pre-existing 5′-truncated *Helitrons* (Supplementary Fig. 4A), (2) insertion right next to another *Helitron* where the 5′-end of one *Helitron* abuts the 3′-end of the other (Supplementary Fig. 4B) and (3) deletion or mutation within the last 30 bp of the 3′-end (Supplementary Fig. 4C). Empty site evidence suggests that these are indeed bona fide insertion events (Supplementary Fig. 4D). Most interestingly, similar to insertion #2 of the pHelRΔ HP transposon (Fig. 6a), we identified one exemplar (Supplementary Fig. 4E), where the *de novo* 3′-end was generated through bypass of the CTAG-3′ sequence in the RTS lacking a palindrome. Thus, bypassing the 3′-end and resulting emergence of *de novo* transposon ends in *Helraiser* transposition (Fig. 6a,b) faithfully recapitulates a natural process.

### Generation of chimeric transcripts by *HelibatN3*

In the *M. lucifugus* genome, promoter sequences from 15 different genes were captured and amplified to 4,690 copies by *Helitrons*^14^. For example, the *HelibatN3* subfamily evolved out of a gene capture event, in which a transposing element picked up a fragment of the *NUBPL* (nucleotide-binding protein-like) gene containing the promoter, coding sequence for six amino acids of the *NUBPL* N terminus and a splice donor sequence (Fig. 6c)^13^. Thus, if a *HelibatN3* element was to jump into an intron of a gene in the correct orientation, it would have the capacity to ectopically express an N-terminally truncated derivative of that gene by splicing between the splice donor sequence in the transposon and the nearest downstream splice acceptor (Fig. 6c).

To demonstrate transcriptional exon-trapping events, we inserted a selectable puro antibiotic resistance gene between the *NUBPL* promoter and the splice donor (Fig. 6c), and mobilized this transposon by the *Helraiser* transposase into the HeLa cell genome. Sequence analysis of complementary DNAs prepared from puro-resistant cells revealed splicing between the transposon-contained splice donor and splice acceptor sites present in human transcripts. These data indicated the capture of exonic sequence downstream of the transposon insertion (Fig. 6c). Furthermore, we also recovered chimeric transcripts, in which the splice donor was apparently spliced to cryptic splice sites in noncoding RNA, resulting in exonization of noncoding genetic information (Fig. 6c).

### *NUBPL*-driven transcripts and their genes in *M. brandtii*

The above data suggest that *HelibatN3* elements act as potent exon traps when mobilized experimentally in HeLa cells. To document the capacity of endogenous *Helitron* transposition to generate novel transcripts *in vivo*, we annotated *Helitron*-captured *NUBPL* promoter-driven transcripts in the bat, *Myotis brandtii*. We found that a *Helitron*-captured *NUBPL* promoter insertion is present within 1-kb upstream of at least one annotated TSS for 23 annotated genes; these insertions are predicted to drive a total of 46 transcripts (FPKM (fragments per kilobase of exon per million fragments mapped) >0.5), three of which have TSS supplied by the insertion (Supplementary Table 3). The majority of these transcripts (43) are predicted to be coding and, in contrast to their parent genes, 35 of the 46 transcripts show some tissue specificity in the tissues examined (FPKM >0.5 in only that tissue; Supplementary Table 3).

Those candidate *NUBPL*-driven transcripts, for which the predicted TSS overlapped with the *Helitron* insertion were considered to be bona fide *NUBPL*-driven transcripts. Three transcripts met this criterion, and belonged to the genes *RINT1* (Supplementary Fig. 5A), *ARMC9* (Supplementary Fig. 5B) and *RNF10* (Supplementary Fig. 5C). Of these, the *RINT1* (kidney) and *RNF10* (constitutively expressed in the tissues examined) transcripts are predicted to be coding (an open reading frame encoding >100 aa is present), and *ARMC9* (brain) noncoding (Supplementary Table 3). In sum, *Helitrons* impact genetic novelty at the transcription level, and *Helraiser* can faithfully recapitulate this biological phenomenon.

## Discussion

We have resurrected an active *Helitron* transposon from the genome of the bat *M. lucifugus*, and used this novel transposon, *Helraiser*, to explore the mechanism and genomic impact of *Helitron* transposition. Consistent with the known properties of other HUH nuclease domains^8^, we detected nuclease activity only on ssDNA fragments derived from *Helraiser's* LTS and RTS *in vitro*. This may indicate that *Helraiser* relies on some cellular process to make ssDNA available for cleavage. For instance, transposition of IS*608*, a well-characterized prokaryotic transposase that encodes an HUH nuclease, is dependent on lagging strand DNA replication to generate ssDNA^39^,^40^. Alternatively, the ssDNA necessary for the initial steps of *Helraiser* transposition could become available through negative supercoiling shown to induce local melting of dsDNA in AT-rich regions^41^,^42^,^43^. In eukaryotic cells, negative supercoiling of DNA occurs upstream of the transcription complex^44^,^45^, and could generate ssDNA patches^46^ required for *Helraiser* transposition. Furthermore, as AT-rich regions can facilitate local DNA melting; perhaps it is not a coincidence that the concensus LTS contains an AT-rich region close to the cleavage site (Supplementary Fig. 1). Both the homology between the *Helraiser* helicase domain and Pif1 (Supplementary Fig. 3A) and the critical requirement of helicase function for transposition (Fig. 2) support a model, in which the role of the helicase domain is to unwind DNA at ssDNA–dsDNA junctions, once ssDNA has been generated at the transposon ends.

Our data suggesting that *Helraiser* transposition proceeds through a circular intermediate defines a crucial distinction when compared with other known eukaryotic DNA transposons. Whether *Helitron* transposition is mechanistically related to some ssDNA-based prokaryotic transposition systems^9^ or to certain ssDNA virus replication processes^47^ remains to be investigated. The lack of local hopping and random distribution of transposon insertions when transposition was initiated from genomic donor loci (Fig. 5) strongly support the idea of episomal transposition intermediates.

**Figure 7:**
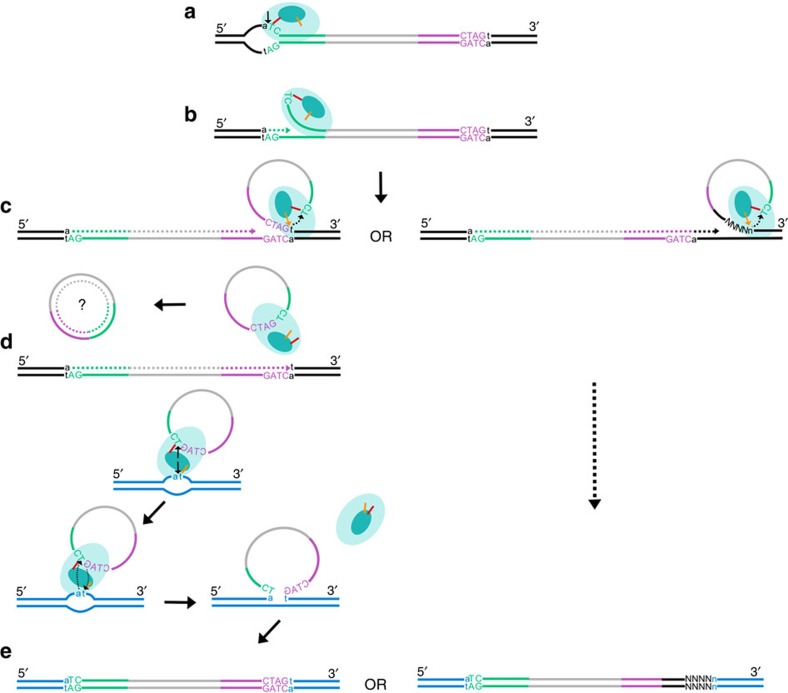
Proposed model of *Helraiser* transposition. (**a**) *Helraiser* transposase (light blue oval) binds the LTS (green) and nicks ssDNA donor site generating a 5′-phosphotyrosine intermediate between the tyrosine residue (orange line) in the HUH nuclease active site (dark blue oval) and the transposon end. (**b**) A free 3′-OH group at the donor site primes DNA synthesis, while the helicase domain unwinds the dsDNA helix in a 5′ to 3′ direction. (**c**) The hairpin structure in the RTS (purple) induces pausing of the helicase required for the recognition and nicking of the CTAG-3′ tetrad at the RTS by the second tyrosine (yellow arrow) of the HUH domain. This generates a free 3′-OH group at the transposon RTS that attacks the first 5′-phosphotyrosine linkage generating a free ssDNA circle. The ssDNA circle is possibly converted into dsDNA circle used for further rounds of transposition. Alternatively, the transposase reads through the RTS and mobilizes host flanking sequences, thereby generating an alternative, *de novo* 3′-end. Further steps in transposition of the canonical transposon and the transposon containing the captured host sequence are identical. (**d**) Two tyrosine residues in the nuclease active site catalyse cleavage of the single-stranded target DNA and the *Helitron* circle, mediating the strand-transfer reaction. (**e**) The single-stranded transposon DNA covalently bound to the target is passively replicated and converted into the double-stranded form during the DNA synthesis phase of the cell cycle, leading to the amplification of the transposon in the host genome and transduction of host genomic sequence.

The following observations are consistent with a modified rolling-circle model of *Helitron* transposition: (1) *Helraiser* transposition requires the LTS, while the RTS is not strictly necessary (Fig. 3); (2) the hairpin appears to be the most important component of the RTS as its deletion or of the whole RTS have similar effects on transposition (Fig. 3); and (3) both transposon truncations and transduction of sequences adjacent to the RTS occur *ex vivo*, and the frequency of these non-canonical transposition events is significantly increased when the hairpin is deleted (Fig. 6). Collectively, the data suggest that the hairpin structure in the RTS plays an important regulatory role in *Helraiser* transposition by serving as a transposition termination signal. Our observations support a ‘read-through' model of capturing DNA sequences flanking the transposon: when the hairpin is missing from the RTS or is not recognized by the transposition machinery, the transposase bypasses the 3′-end of the transposon and finds an alternative transposition terminator sequence further downstream, resulting in transduction of the flanking host sequence^25^ (Fig. 7).

The relatively loose functional definition of the RTS is most likely the core reason why *Helitrons* can efficiently transduce downstream host genomic sequences. Gene capture may contribute to the emergence and diversification of novel *Helitron* families and to the generation of novel cellular transcripts. For example, the captured *NUBPL* gene fragment, when mobilized by the *Helraiser* transposase into the genome of human cells, gives rise to novel coding and noncoding transcripts by imposed transcription and splicing (Fig. 6c). We identified several *HelibatN3* insertions that drive transcription of cellular genes (Supplementary Table 3), and identified transcripts that initiate within the *NUBPL* insertion. All of these bona fide *NUBPL*-driven transcripts were N-terminally truncated and had exonized noncoding sequence, most often resulting in a novel 5′-UTR (Supplementary Fig. 5; Supplementary Table 3), as seen with some of the *Helraiser*-catalysed insertions *ex vivo* (Fig. 6c).

Transposable elements have been shaping genome structure and function for millions of years, and have exerted a strong influence on the evolutionary trajectory of their hosts (reviewed in ref. 48). The most prominent agents documented to provide alternative promoters, enhancer elements, polyadenylation signals and splice sites are retrotransposons. In addition, it has been shown that ∼1,000 cellular gene fragments had been captured by cut-and-paste Pack-MULE DNA transposons in the rice genome, suggesting that these transposons might have played a role in the evolution of genes in plants^49^. It appears that *Helitrons* also have a profound potential to generate genome variation. Indeed, ∼60% of maize *Helitrons* were found to carry captured gene fragments, adding up to tens of thousands of gene fragments disseminated across the maize genome by *Helitron* transposition^31^. Although most captured gene fragments are apparently undergoing random drift in maize, ∼4% of them are estimated to be under purifying selection, suggesting beneficial effects for the host. Thus, the molecular mechanism of 3′-transduction and subsequent, genome-wide dissemination of captured gene fragments or entire genes by copy-and-paste transposition uniquely positions *Helitrons* as powerful genome shuffling agents with wide-reaching biological consequences.

## Methods

### Constructs and PCRs

Detailed cloning procedures of transposon and transposase expression vectors as well as primer sequences for PCRs are provided in Supplementary Notes and Supplementary Table 4.

### Cells and transfection

HeLa cells (2 × 10^5^, American Type Culture Collection) were seeded onto six-well plates 1 day before transfection. Two microlitres of jetPRIME transfection reagent (Polyplus Transfection) and 200 μl of jetPRIME buffer were used to transfect 1 μg of DNA (each transfection reaction contained 500 ng transposon donor and 500 ng transposase helper or pBluescript vector (Stratagene). Forty-eight hours after transfection, a fraction of the transfected cells (10 or 20%) was replated on 100-mm dishes and selected for transposon integration (2 μg ml^−1^ puro or 2 μg ml^−1^ puro and 1.4 mg ml^−1^ G418). After 2–3 weeks of selection, colonies were either picked or fixed in 4% paraformaldehyde in PBS and stained with methylene blue in PBS for colony counting and analysis.

### Insertion site and copy-number analysis by splinkerette PCR

Transposon copy numbers were determined by splinkerette PCR as detailed in Supplementary Notes.

### Circle detection assay

Low-molecular-weight DNA was isolated from transfected HeLa cells and used in a modified inverse PCR protocol to detect *Helitron* circles. Further details are provided in Supplementary Notes.

### *Helraiser* retransposition in HeLa cells

Cells expressing the *Helraiser* transposase were enriched by repeatedly transfecting the HeLa-derived transposon donor H1 cell line containing three unambiguously mapped *Helraiser* insertions with the pCHelRGFP helper plasmid and sorting green fluorescent protein-positive cells. We then subjected the pooled DNA of the enriched cell population to high-throughput sequencing of transposon insertion sites. Further details are provided in Supplementary Notes.

### Genome-wide insertion site analysis

HeLa cells were transfected as previously described with pCHelR and pHelR. Three weeks post transfection, puro-resistant colonies were pooled and genomic DNA isolated. DNA sequences flanking the transposon ends were mapped against the human genome (hg19) with Bowtie^50^ allowing up to one mismatch. Only uniquely mapped reads matching to the genome without error were kept. Redundant reads mapping to the same genomic location were merged together. We discarded all integrations into genomic locations matching to the last four bases of the transposon end, because these sites could also be mispriming artifacts. Further details are provided in Supplementary Notes.

### Protein expression and purification

Point mutations were made using the QuikChange site-directed mutagenesis method (Agilent). Baculovirus production and protein expression were performed by the Protein Expression Laboratory at the National Cancer Institute as detailed in Supplementary Notes.

### Cleavage assay and sequencing of cleavage products

DNA cleavage was measured using 6-FAM-labelled oligonucleotides (BioTeZ Berlin-Buch GMBH). Reactions generally consisted of 500 nM DNA substrate and 500 nM protein in buffer (50 mM Tris pH 7.5, 100 mM NaCl, 0.5 mM ETDA and 1 mM TCEP) with or without 5 mM MnCl_2_. Further details are provided in Supplementary Notes.

### Electrophoretic mobility shift assay (EMSA)

Binding of the *Helraiser* transposase to various DNA oligonucleotides was measured by EMSA using 6% TBE gels (Invitrogen). Purified protein at 15–250 nM was incubated for 30 min at room temperature in binding buffer (50 mM Tris pH 7.5, 100 mM NaCl, 10 mM MgCl_2_, 0.5 mM EDTA, 1 mM TCEP) with 50 nM 6-FAM-labelled oligonucleotides. To test whether the addition of a nonhydrolyzable ATP analogue could lock *Helraiser* helicase domain into an active conformation and facilitate DNA binding, 1 mM AMP–PNP was added to some of the binding reactions. After addition of DNA gel loading solution (Quality Biological, INC), samples were run on 6% TBE gels and visualized.

## Additional information

**How to cite this article:** Grabundzija, I. *et al*. A Helitron transposon reconstructed from bats reveals a novel mechanism of genome shuffling in eukaryotes. *Nat. Commun.* 7:10716 doi: 10.1038/ncomms10716 (2016).

## Supplementary Material

Supplementary Information Supplementary Figures 1-5, Supplementary Table 1-4, Supplementary Notes 1-18 and Supplementary References

## References

[b1] Kapitonov V. V. & Jurka J. Helitrons on a roll: eukaryotic rolling-circle transposons. Trends Genet. 23, 521–529 (2007). 17850916 10.1016/j.tig.2007.08.004

[b2] Thomas J. & Pritham E. J. Helitrons, the eukaryotic rolling-circle transposable elements. Microbiol. Spectr. 3, 893–926 (2015). 10.1128/microbiolspec.MDNA3-0049-2014 26350323

[b3] Dyda F. . Crystal structure of the catalytic domain of HIV-1 integrase: similarity to other polynucleotidyl transferases. Science 266, 1981–1986 (1994). 7801124 10.1126/science.7801124

[b4] Kapitonov V. V. & Jurka J. Rolling-circle transposons in eukaryotes. Proc. Natl Acad. Sci. USA 98, 8714–8719 (2001). 11447285 10.1073/pnas.151269298 PMC37501

[b5] Ilyina T. V. & Koonin E. V. Conserved sequence motifs in the initiator proteins for rolling circle DNA replication encoded by diverse replicons from eubacteria, eucaryotes and archaebacteria. Nucleic Acids Res. 20, 3279–3285 (1992). 1630899 10.1093/nar/20.13.3279 PMC312478

[b6] Koonin E. V. & Ilyina T. V. Computer-assisted dissection of rolling circle DNA replication. Biosystems 30, 241–268 (1993). 8374079 10.1016/0303-2647(93)90074-m

[b7] van Mansfeld A. D., van Teeffelen H. A., Baas P. D. & Jansz H. S. Two juxtaposed tyrosyl-OH groups participate in phi X174 gene A protein catalysed cleavage and ligation of DNA. Nucleic Acids Res. 14, 4229–4238 (1986). 2940511 10.1093/nar/14.10.4229 PMC339857

[b8] Chandler M. . Breaking and joining single-stranded DNA: the HUH endonuclease superfamily. Nat. Rev. Microbiol.y 11, 525–538 (2013). 10.1038/nrmicro3067 PMC6493337 23832240

[b9] del Pilar Garcillan-Barcia M., Bernales I., Mendiola M. V. & de la Cruz F. Single-stranded DNAintermediates in IS91 rolling-circle transposition. Mol. Microbiol. 39, 494–501 (2001). 11136468 10.1046/j.1365-2958.2001.02261.x

[b10] Garcillan-Barcia M. P. & de la Cruz F. Distribution of IS91 family insertion sequences in bacterial genomes: evolutionary implications. FEMS Microbiol. Ecol. 42, 303–313 (2002). 19709290 10.1111/j.1574-6941.2002.tb01020.x

[b11] Mendiola M. V., Bernales I. & de la Cruz F. Differential roles of the transposon termini in IS91 transposition. Proc. Natl Acad. Sci. USA 91, 1922–1926 (1994). 8127907 10.1073/pnas.91.5.1922 PMC43276

[b12] Mendiola M. V. & de la Cruz F. IS91 transposase is related to the rolling-circle-type replication proteins of the pUB110 family of plasmids. Nucleic Acids Res. 20, 3521 (1992). 1321417 10.1093/nar/20.13.3521 PMC312521

[b13] Pritham E. J. & Feschotte C. Massive amplification of rolling-circle transposons in the lineage of the bat Myotis lucifugus. Proc. Natl Acad. Sci. USA 104, 1895–1900 (2007). 17261799 10.1073/pnas.0609601104 PMC1794268

[b14] Thomas J., Phillips C. D., Baker R. J. & Pritham E. J. Rolling-circle transposons catalyze genomic innovation in a Mammalian lineage. Genome Biol. Evol. 6, 2595–2610 (2014). 25223768 10.1093/gbe/evu204 PMC4224331

[b15] Thomas J., Sorourian M., Ray D., Baker R. J. & Pritham E. J. The limited distribution of Helitrons to vesper bats supports horizontal transfer. Gene 474, 52–58 (2011). 21193022 10.1016/j.gene.2010.12.007

[b16] Coates B. S., Hellmich R. L., Grant D. M. & Abel C. A. Mobilizing the genome of Lepidoptera through novel sequence gains and end creation by non-autonomous Lep1 Helitrons. DNA Res. 19, 11–21 (2012). 22086996 10.1093/dnares/dsr038 PMC3276263

[b17] Du C., Fefelova N., Caronna J., He L. & Dooner H. K. The polychromatic Helitron landscape of the maize genome. Proc. Natl Acad. Sci. USA 106, 19916–19921 (2009). 19926866 10.1073/pnas.0904742106 PMC2785267

[b18] Lal S. K., Giroux M. J., Brendel V., Vallejos C. E. & Hannah L. C. The maize genome contains a helitron insertion. Plant Cell 15, 381–391 (2003). 12566579 10.1105/tpc.008375 PMC141208

[b19] Xiong W., He L., Lai J., Dooner H. K. & Du C. HelitronScanner uncovers a large overlooked cache of Helitron transposons in many plant genomes. Proc. Natl Acad. Sci. USA 111, 10263–10268 (2014). 24982153 10.1073/pnas.1410068111 PMC4104883

[b20] Morgante M. . Gene duplication and exon shuffling by helitron-like transposons generate intraspecies diversity in maize. Nat. Genet. 37, 997–1002 (2005). 16056225 10.1038/ng1615

[b21] Dong Y. . Structural characterization of helitrons and their stepwise capturing of gene fragments in the maize genome. BMC Genomics 12, 609 (2011). 22177531 10.1186/1471-2164-12-609 PMC3288121

[b22] Toleman M. A., Bennett P. M. & Walsh T. R. ISCR elements: novel gene-capturing systems of the 21st century? Microbiol. Mol. Biol. Rev. 70, 296–316 (2006). 16760305 10.1128/MMBR.00048-05 PMC1489542

[b23] Yassine H. . Experimental evidence for IS1294b-mediated transposition of the blaCMY-2 cephalosporinase gene in Enterobacteriaceae. J. Antimicrob. Chemother. 70, 697–700 (2015). 25428924 10.1093/jac/dku472

[b24] Brunner S., Pea G. & Rafalski A. Origins, genetic organization and transcription of a family of non-autonomous helitron elements in maize. Plant J. 43, 799–810 (2005). 16146520 10.1111/j.1365-313X.2005.02497.x

[b25] Feschotte C. & Wessler S. R. Treasures in the attic: rolling circle transposons discovered in eukaryotic genomes. Proc. Natl Acad. Sci. USA 98, 8923–8924 (2001). 11481459 10.1073/pnas.171326198 PMC55346

[b26] Tempel S., Nicolas J., El Amrani A. & Couee I. Model-based identification of Helitrons results in a new classification of their families in Arabidopsis thaliana. Gene 403, 18–28 (2007). 17889452 10.1016/j.gene.2007.06.030

[b27] Mates L. . Molecular evolution of a novel hyperactive Sleeping Beauty transposase enables robust stable gene transfer in vertebrates. Nat. Genet. 41, 753–761 (2009). 19412179 10.1038/ng.343

[b28] Bird L. E., Subramanya H. S. & Wigley D. B. Helicases: a unifying structural theme? Curr. Opin. Struct. Biol. 8, 14–18 (1998). 9519291 10.1016/s0959-440x(98)80004-3

[b29] Han M. J. . Identification and evolution of the silkworm helitrons and their contribution to transcripts. DNA Res. 20, 471–484 (2013). 23771679 10.1093/dnares/dst024 PMC3789558

[b30] Yang L. & Bennetzen J. L. Structure-based discovery and description of plant and animal Helitrons. Proc. Natl Acad. Sci. USA 106, 12832–12837 (2009). 19622734 10.1073/pnas.0905563106 PMC2722332

[b31] Yang L. & Bennetzen J. L. Distribution, diversity, evolution, and survival of Helitrons in the maize genome. Proc. Natl Acad. Sci. USA 106, 19922–19927 (2009). 19926865 10.1073/pnas.0908008106 PMC2785268

[b32] Harrow J. . GENCODE: the reference human genome annotation for The ENCODE Project. Genome Res. 22, 1760–1774 (2012). 22955987 10.1101/gr.135350.111 PMC3431492

[b33] Andersson R. . An atlas of active enhancers across human cell types and tissues. Nature 507, 455–461 (2014). 24670763 10.1038/nature12787 PMC5215096

[b34] Guelen L. . Domain organization of human chromosomes revealed by mapping of nuclear lamina interactions. Nature 453, 948–951 (2008). 18463634 10.1038/nature06947

[b35] Carlson C. M. . Transposon mutagenesis of the mouse germline. Genetics 165, 243–256 (2003). 14504232 10.1093/genetics/165.1.243 PMC1462753

[b36] Fischer S. E., Wienholds E. & Plasterk R. H. Regulated transposition of a fish transposon in the mouse germ line. Proc. Natl Acad. Sci. USA 98, 6759–6764 (2001). 11381141 10.1073/pnas.121569298 PMC34426

[b37] Luo G., Ivics Z., Izsvak Z. & Bradley A. Chromosomal transposition of a Tc1/mariner-like element in mouse embryonic stem cells. Proc. Natl Acad. Sci. USA 95, 10769–10773 (1998). 9724779 10.1073/pnas.95.18.10769 PMC27970

[b38] Tower J., Karpen G. H., Craig N. & Spradling A. C. Preferential transposition of Drosophila P elements to nearby chromosomal sites. Genetics 133, 347–359 (1993). 8382177 10.1093/genetics/133.2.347 PMC1205324

[b39] Ton-Hoang B. . Transposition of ISHp608, member of an unusual family of bacterial insertion sequences. EMBO J. 24, 3325–3338 (2005). 16163392 10.1038/sj.emboj.7600787 PMC1224677

[b40] Ton-Hoang B. . Single-stranded DNA transposition is coupled to host replication. Cell 142, 398–408 (2010). 20691900 10.1016/j.cell.2010.06.034 PMC2919506

[b41] Dayn A., Malkhosyan S. & Mirkin S. M. Transcriptionally driven cruciform formation in vivo. Nucleic Acids Res. 20, 5991–5997 (1992). 1461732 10.1093/nar/20.22.5991 PMC334465

[b42] Krasilnikov A. S., Podtelezhnikov A., Vologodskii A. & Mirkin S. M. Large-scale effects of transcriptional DNA supercoiling in vivo. J. Mol. Biol. 292, 1149–1160 (1999). 10512709 10.1006/jmbi.1999.3117

[b43] Strick T. R., Allemand J. F., Bensimon D. & Croquette V. Behavior of supercoiled DNA. Biophys. J. 74, 2016–2028 (1998). 9545060 10.1016/S0006-3495(98)77908-1 PMC1299542

[b44] Liu L. F. & Wang J. C. Supercoiling of the DNA template during transcription. Proc.Natl Acad. Sci. USA 84, 7024–7027 (1987). 2823250 10.1073/pnas.84.20.7024 PMC299221

[b45] Rahmouni A. R. & Wells R. D. Direct evidence for the effect of transcription on local DNA supercoiling in vivo. J. Mol. Biol. 223, 131–144 (1992). 1731065 10.1016/0022-2836(92)90721-u

[b46] Parsa J. Y. . Negative supercoiling creates single-stranded patches of DNA that are substrates for AID-mediated mutagenesis. PLoS Genet. 8, e1002518 (2012). 22346767 10.1371/journal.pgen.1002518 PMC3276561

[b47] Faurez F., Dory D., Grasland B. & Jestin A. Replication of porcine circoviruses. Virol. J. 6, 60 (2009). 19450240 10.1186/1743-422X-6-60 PMC2690592

[b48] Feschotte C. Transposable elements and the evolution of regulatory networks. Nat. Rev. Genet. 9, 397–405 (2008). 18368054 10.1038/nrg2337 PMC2596197

[b49] Jiang N., Bao Z., Zhang X., Eddy S. R. & Wessler S. R. Pack-MULE transposable elements mediate gene evolution in plants. Nature 431, 569–573 (2004). 15457261 10.1038/nature02953

[b50] Langmead B., Trapnell C., Pop M. & Salzberg S. L. Ultrafast and memory-efficient alignment of short DNA sequences to the human genome. Genome Biol. 10, R25 (2009). 19261174 10.1186/gb-2009-10-3-r25 PMC2690996

[b51] Zuker M. Mfold web server for nucleic acid folding and hybridization prediction. Nucleic Acids Res. 31, 3406–3415 (2003). 12824337 10.1093/nar/gkg595 PMC169194

