# Supplementary material for: A Helitron transposon reconstructed from bats reveals a novel mechanism of genome shuffling in eukaryotes
Source: Nat Commun. 2016 Mar 2;7:10716. doi: 10.1038/ncomms10716 (PMC4778049; doi:10.1038/ncomms10716)
Supplement: Supplementary Information — Supplementary Figures 1-5, Supplementary Table 1-4, Supplementary Notes 1-18 and Supplementary References [file ncomms10716-s1.pdf]

Supplementary Information

Supplementary Figures

Helibat1 (Helraiser) complete consensus sequence

TCCTATATAATAAAAGAGAAACATGCAAATTGACCATCCCTCCGCTACGCTCAAGCCACGCCCACCAGCCAATCAGAAGTGACTATGCAAATTAACCCAACAAAGATGGCAGTTAAATTTGCATACGCAGGT
GTCAAGCGCCCCAGGAGGCAACGGCGGCCGCGGGCTCCCAGGACCTTCGCTGGCCCCGGGAGGCGAGGCCGGCCGCGCCTAGCCACACCCGCGGGCTCCCGGGACCTTCGCCAGCAGAGAGCAGAGCGGGAG
AGCGGGCGGAGAGCGGGAGGTTTGGAGGACTTGGCAGAGCAGGAGGCCGCTGGACATAGAGCAGAGCGAGAGAGAGGGTGGCTTGGAGGGCGTGGCTCCCTCTGTCACCCCAGCTTCCTCATCACAGCTGTG
GAAACTGACAGCAGGGAGGAGGAAGTCCCACCCCCACAGAATCAGCCAGAATCAGCCGTTGGTCAGACAGCTCTCAGCGGCCTGACAGCCAGGACTCTCATTCACCTGCATCTCAGACCGTGACAGTAGAGA
GGTGGGACTATGTCTAAAGAACAACTGTTGATACAACGTAGCTCTGCAGCCGAAAGATGCCGGCGTTATCGACAGAAAATGTCTGCAGAGCAACGTGCGTCTGATCTTGAAAGAAGGCGGCGCCTGCAACAG
AATGTATCTGAAGAGCAGCTACTGGAAAAACGTCGCTCTGAAGCCGAAAAACAGCGGCGTCATCGACAGAAAATGTCTAAAGACCAACGTGCCTTTGAAGTTGAAAGAAGGCGGTGGCGACGACAGAATATG
TCTAGAGAACAGTCATCAACAAGTACTACCAATACCGGTAGGAACTGCCTTCTCAGCAAAAATGGAGTACATGAGGATGCAATTCTCGAACATAGTTGTGGTGGAATGACTGTTCGATGTGAATTTTGCCTA
TCACTAAATTTCTCTGATGAAAAACCATCCGATGGGAAATTTACTCGATGTTGTAGCAAAGGGAAAGTCTGTCCAAATGATATACATTTTCCAGATTACCCGGCATATTTAAAAAGATTAATGACAAACGAA
GATTCTGACAGTAAAAATTTCATGGAAAATATTCGTTCCATAAATAGTTCTTTTGCTTTTGCTTCCATGGGTGCAAATATTGCATCGCCATCAGGATATGGGCCATACTGTTTTAGAATACACGGACAAGTT
TATCACCGTACTGGAACTTTACATCCTTCGGATGGTGTTTCTCGGAAGTTTGCTCAACTCTATATTTTGGATACAGCCGAAGCTACAAGTAAAAGATTAGCAATGCCAGAAAACCAGGGCTGCTCAGAAAGA
CTCATGATCAACATCAACAACCTCATGCATGAAATAAATGAATTAACAAAATCGTACAAGATGCTACATGAGGTAGAAAAGGAAGCCCAATCTGAAGCAGCAGCAAAAGGTATTGCTCCCACAGAAGTAACA
ATGGCGATTAAATACGATCGTAACAGTGACCCAGGTAGATATAATTCTCCCCGTGTAACCGAGGTTGCTGTCATATTCAGAAACGAAGATGGAGAACCTCCTTTTGAAAGGGACTTGCTCATTCATTGTAAA
CCAGATCCCAATAATCCAAATGCCACTAAAATGAAACAAATCAGTATCCTGTTTCCTACATTAGATGCAATGACATATCCTATTCTTTTTCCACATGGTGAAAAAGGCTGGGGAACAGATATTGCATTAAGA
CTCAGAGACAACAGTGTAATCGACAATAATACTAGACAAAATGTAAGGACACGAGTCACACAAATGCAGTATTATGGATTTCATCTCTCTGTGCGGGACACGTTCAATCCTATTTTAAATGCAGGAAAATTA
ACTCAACAGTTTATTGTGGATTCATATTCAAAAATGGAGGCCAATCGGATAAATTTCATCAAAGCAAACCAATCTAAGTTGAGAGTTGAAAAATATAGTGGTTTGATGGATTATCTCAAATCTAGATCTGAA
AATGACAATGTGCCGATTGGTAAAATGATAATACTTCCATCATCTTTTGAGGGTAGTCCCAGAAATATGCAGCAGCGATATCAGGATGCTATGGCAATTGTAACGAAGTATGGCAAGCCCGATTTATTCATA
ACCATGACATGCAACCCCAAATGGGCAGATATTACAAACAATTTACAACGCTGGCAAAAAGTTGAAAACAGACCTGACTTGGTAGCCAGAGTTTTTAATATTAAGCTGAATGCTCTTTTAAATGATATATGT
AAATTCCATTTATTTGGCAAAGTAATAGCTAAAATTCATGTCATTGAATTTCAGAAACGCGGACTGCCTCACGCTCACATATTATTGATATTAGATAGTGAGTCCAAATTACGTTCAGAAGATGACATTGAC
CGTATAGTTAAGGCAGAAATTCCAGATGAAGACCAGTGTCCTCGACTTTTTCAAATTGTAAAATCAAATATGGTACATGGACCATGTGGAATACAAAATCCAAATAGTCCATGTATGGAAAATGGAAAATGT
TCAAAGGGATATCCAAAAGAATTTCAAAATGCGACCATTGGAAATATTGATGGATATCCCAAATACAAACGAAGATCTGGTAGCACCATGTCTATTGGAAATAAAGTTGTCGATAACACTTGGATTGTCCCT
TATAACCCGTATTTGTGCCTTAAATATAACTGTCATATAAATGTTGAAGTCTGTGCATCAATTAAAAGTGTCAAATATTTATTTAAATACATCTATAAAGGGCACGATTGTGCAAATATTCAAATTTCTGAA
AAAAATATTATCAATCATGACGAAGTACAGGACTTCATTGACTCCAGGTATGTGAGCGCTCCTGAGGCTGTTTGGAGACTTTTTGCAATGCGAATGCATGACCAATCTCATGCAATCACAAGATTAGCTATT
CATTTGCCAAATGATCAGAATTTGTATTTTCATACCGATGATTTTGCTGAAGTTTTAGATAGGGCTAAAAGGCATAACTCGACTTTGATGGCTTGGTTCTTATTGAATAGAGAAGATTCTGATGCACGTAAT
TATTATTATTGGGAGATTCCACAGCATTATGTGTTTAATAATTCTTTGTGGACAAAACGCCGAAAGGGTGGGAATAAAGTATTAGGTAGACTGTTCACTGTGAGCTTTAGAGAACCAGAACGATATTACCTT
AGACTTTTGCTTCTGCATGTAAAAGGTGCGATAAGTTTTGAGGATCTGCGAACTGTAGGAGGTGTAACTTATGATACATTTCATGAAGCTGCTAAACACCGAGGATTATTACTTGATGACACTATCTGGAAA
GATACGATTGACGATGCAATCATCCTTAATATGCCCAAACAACTACGGCAACTTTTTGCATATATATGTGTGTTTGGATGTCCTTCTGCTGCAGACAAATTATGGGATGAGAATAAATCTCATTTTATTGAA
GATTTCTGTTGGAAATTACACCGAAGAGAAGGTGCCTGTGTGAACTGTGAAATGCATGCCCTTAACGAAATTCAGGAGGTATTCACATTGCATGGAATGAAATGTTCACATTTCAAACTTCCGGACTATCCT
TTATTAATGAATGCAAATACATGTGATCAATTGTACGAGCAACAACAGGCAGAGGTTTTGATAAATTCTCTGAATGATGAACAGTTGGCAGCCTTTCAGACTATAACTTCAGCCATCGAAGATCAAACTGTA
CACCCCAAATGCTTTTTCTTGGATGGTCCAGGTGGTAGTGGAAAAACATATCTGTATAAAGTTTTAACACATTATATTAGAGGTCGTGGTGGTACTGTTTTACCCACAGCATCTACAGGAATTGCTGCAAAT
TTACTTCTTGGTGGAAGAACCTTTCATTCCCAATATAAATTACCAATTCCATTAAATGAAACTTCAATTTCTAGACTCGATATAAAGAGTGAAGTTGCTAAAACCATTAAAAAGGCCCAACTTCTCATTATT
GATGAATGCACCATGGCATCCAGTCATGCTATAAACGCCATAGATAGATTACTAAGAGAAATTATGAATTTGAATGTTGCATTTGGTGGGAAAGTTCTCCTTCTCGGAGGGGATTTTCGACAATGTCTCAGT
ATTGTACCACATGCTATGCGATCGGCCATAGTACAAACGAGTTTAAAGTACTGTAATGTTTGGGGATGTTTCAGAAAGTTGTCTCTTAAAACAAATATGAGATCAGAGGATTCTGCTTATAGTGAATGGTTA
GTAAAACTTGGAGATGGCAAACTTGATAGCAGTTTTCATTTAGGAATGGATATTATTGAAATCCCCCATGAAATGATTTGTAACGGATCTATTATTGAAGCTACCTTTGGAAATAGTATATCTATAGATAAT
ATTAAAAATATATCTAAACGTGCAATTCTTTGTCCAAAAAATGAGCATGTTCAAAAATTAAATGAAGAAATTTTGGATATACTTGATGGAGATTTTCACACATATTTGAGTGATGATTCCATTGATTCAACA
GATGATGCTGAAAAGGAAAATTTTCCCATCGAATTTCTTAATAGTATTACTCCTTCGGGAATGCCGTGTCATAAATTAAAATTGAAAGTGGGTGCAATCATCATGCTATTGAGAAATCTTAATAGTAAATGG
GGTCTTTGTAATGGTACTAGATTTATTATCAAAAGATTACGACCTAACATTATCGAAGCTGAAGTATTAACAGGATCTGCAGAGGGAGAGGTTGTTCTGATTCCAAGAATTGATTTGTCCCCATCTGACACT
GGCCTCCCATTTAAATTAATTCGAAGACAGTTTCCCGTGATGCCAGCATTTGCGATGACTATTAATAAATCACAAGGACAAACTCTAGACAGAGTAGGAATATTCCTACCTGAACCCGTTTTCGCACATGGT
CAGTTATATGTTGCTTTCTCTCGAGTTCGAAGAGCATGTGACGTTAAAGTTAAAGTTGTAAATACTTCATCACAAGGGAAATTAGTCAAGCACTCTGAAAGTGTTTTTACTCTTAATGTGGTATACAGGGAG
ATATTAGAATAAGTTTAATCACTTTATCAGTCATTGTTTGCATCAATGTTGTTTTTATATCATGTTTTTGTTGTTTTTATATCATGTCTTTGTTGTTGTTATATCATGTTGTTATTGTTATTTATTAATAA
ATTTATGTATTATTTTCATATACATTTTACTCATTTCCTTTCATCTCTCACACTTCTATTATAGAGAAAGGGCAAATAGCAATATTAAAATATTTCCTCTAATTAATTCCCTTTCAATGTGCACGAATTTCG
TGCACCGGGCCA**CTAG**

Helibat1

TCCTATATAATAAAAGAGAAACATGCAAATTGACCATCCCTCCGCTACGCTCAAGCCACGCCCACCAGCCAATCAGAAGTGACTATGCAAATTAACCCAACAAAGATGGCAGTTAAATTTGCATACGCAGGT
GTCAAGCGCCCCAGGAGGAAATTTATGTATTATTTTCATATACATTTTACTCATTTCCTTTCATCTCTCACACTTCTATTATAGAGAAAGGGCAAATAGCAATATTAAAATATTTCCTCTAATTAATTCCCT
TTCAATGTGCACGAATTTCGTGCACCGGGCCA**CTAG**

HelibatN1

TCCTATATAATAAAAGAGAAACATGCAAATTAGCCGTCCCTCTGCTACGCTCAAGCCACGCCCACAAGCCACGCCCACCAGCCAATCAGAGTGACTATGCAAATTAACCTGACAAAGATGGCGGTTAATTTG
CATACGCAGGTGTCAAGCAAAGTAATAGCTAAAATTCATGTCATTGAATTTCAGAAACGCGGACTGCCTCACGCTCACATATTATTGATATTAGATAGTGAGTCCAAATTACGTTCCTCTAATTAATTCCCT
TTCAATGTGCACGAATTTCGTGCACCAGGCTA**CTAG**

HelibatN2

TCTACTTATATAAAAACCCTGGGTGTAACATCACGTCCAGAAGCGCGACCAACCGGAAGGAAGTCAGTCCTGCAGGGGTCGTCTTGGAAACGGCTGCGCCCTCCCCCAGCTGTTTCCCCGGAGGGCGAGGTT
TCAGGCAGGAACCCGCCCAAATTTATATATTATTTTCATATACATTTTACTAATTTCCTTTCATCTCTCACACTTCTATTATAGAGAAAGGGCAAATAGCAATATTAAAATATCTCCGCTAATTAATTCCCT
TTTAATGTGCACGAATTTCGTGCACCGGGCTA**CTAG**

HelibatN3

TCTATATATATAAAAGGCTAAGTGTCCATCCAACCGGTAGCTATGATGCACACTGACCACCAGGGGGCAGATGCTCAATGCAGGAGCTGCCATGATGTGCACTGGCCATTTAAAAATAAACGTGGGCTGGAA
AAAGTTTAGCTAAATCAGAAAGCAGGTCTAATTAAGCAAGTTTATTCTATATCTATAAAAGGCTAAGTTGACTCGTGCATGCGCGATACATATAAAGCTCTCGCTGGCGCCAATCGCACACGTGTGTTTCGA
TCTGTCATTGTCGATCATGAATTTGGTTGACACTTCTATTATAGAGAAAGGGCAAATAGCGATATTAAAATATTTCTTCTAATTAATTCCTTTCAATGTGCACGAATCTGTGCACCGGGCCA**CTAG**

Transposase coding sequences
Left terminal sequences (LTS)
Right terminal sequences (RTS)
Hairpin

Supplementary Figure 1. DNA sequences. The complete DNA sequence of the consensus *Helibat1* (*Helraiser*) transposon, and the consensus left terminal and right terminal sequences of autonomous and non-autonomous transposons that were used in the transposon donor constructs. The 5'-TC and CTAG-3' terminal sequences are typed in bold.

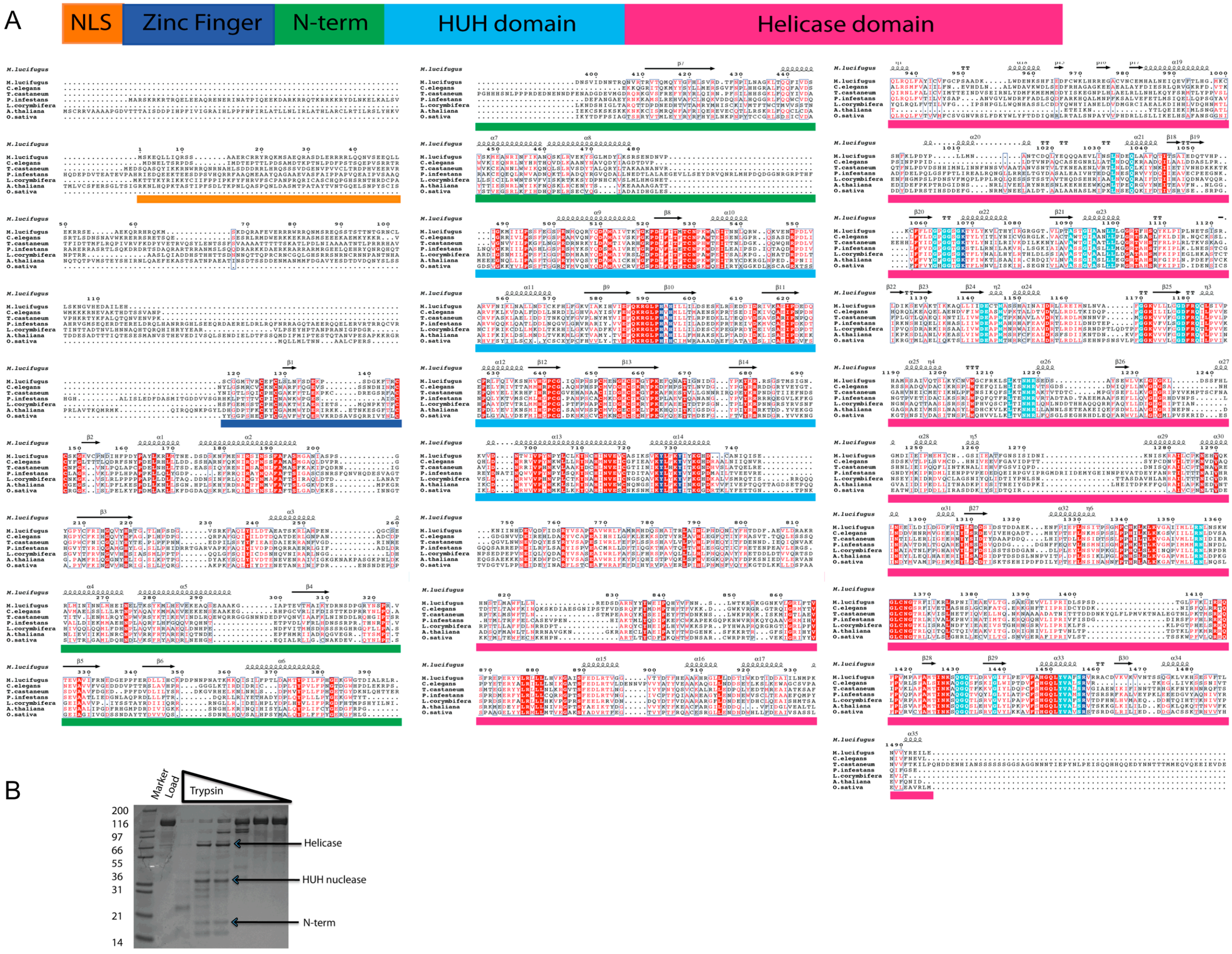

Supplementary Figure 2. Protein sequence alignment and domain mapping. A) Overall protein sequence alignment of *Helraiser* from *M. lucifugus* with *Helitron* transposase sequences from *C. elegans*, *T. castaneum, P. infestans*, *L. corymbifera*, *A. thaliana* and *O. sativa*. Secondary structural features show beta strands as arrows and helices as spirals. The domains determined by trypsin digest are represented as colored bars below the sequence. Predicted nuclear localization signal and zinc-finger motifs are indicated by orange and violet bars below the sequence, respectively. Mutated catalytic residues are highlighted in blue, helicase motifs in cyan and identical residues in red. **B)** SDS-PAGE analysis of the purified *Helraiser* digest by increasing amounts of trypsin. N-terminal sequencing identified the helicase fragment encompassing the amino acids 811-1496, HUH nuclease fragment containing amino acids 491-745 and N-terminal fragment spanning the amino acids 251 to 481.

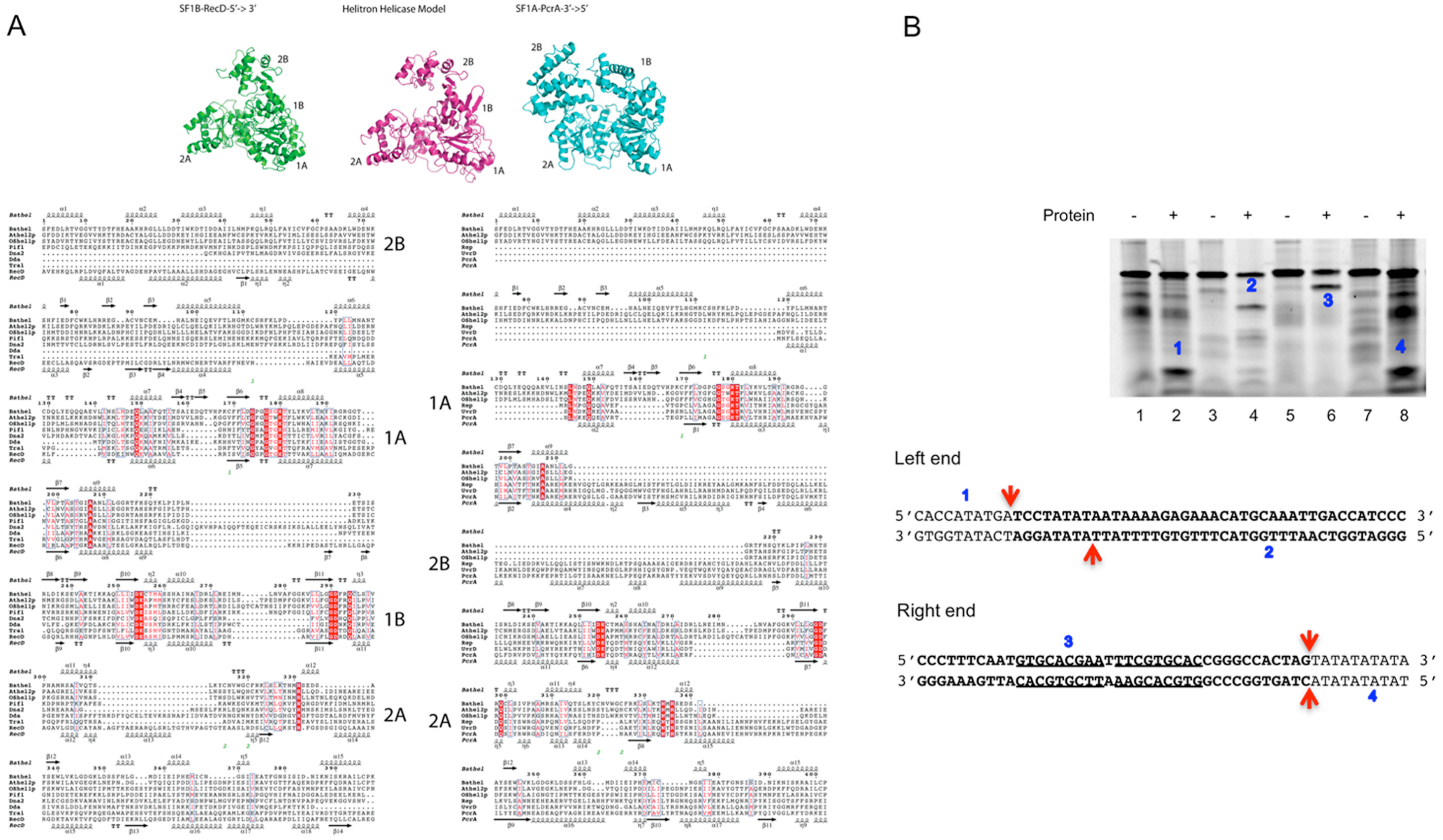

Supplementary Figure 3. Structural and functional properties of *Helraiser* transposase domains. The *Helraiser* helicase domain aligns with the SF1B subfamily group members and is closely related to Pif1 helicases, a family of helicases involved in homologous recombination and the resolution of stalled replication forks[1, 2, 3], a previously noted feature of *Helitrons*[4]. **A)** Helicase sequence alignment. Top: ribbon models of RecD helicase from *E. coli, Helitron* helicase and PcrA helicase from *G. stearothermophilus* are shown in green, purple and cyan, respectively. The four domains of each helicase are labeled. Bottom: overall protein sequence alignment of *Helitron* helicases from *M. lucifugus* (*Helraiser*), *A. thaliana* and *O. sativa* with SF1B helicases [(Pif1 (*S. cerevisiae*), Dna2 (*S. cerevisiae*), Dda (*E. phage T4*), TraI (*E. coli*) and RecD (*E. coli*)] on the left and SF1A helicases [Rep (*E.coli*), UvrD (*B. subtilis ssp. subtilis* str. 168) and PcrA (*G. stearothermophilus*)] on the rigt. Secondary structural features show beta sheets as arrows and helices as spirals. Conserved SF1 helicase motifs are highlighted in red. **B)** *In vitro* cleavage ssDNA of 5'- or 3'-terminus top and bottom strand. 15% TBE-UREA gel of 5' FAM-labeled oligonucleotides cleavage by *Helraiser* transposase. Schematic of DNA on the right depicts the four ssDNA substrates, 5'- and 3'-terminus sequence is in bold, flanking sequence in regular script, and 3'-hairpin is underlined. The red arrows show the cleavage sites, and blue numbers the ssDNA fragments sequenced.

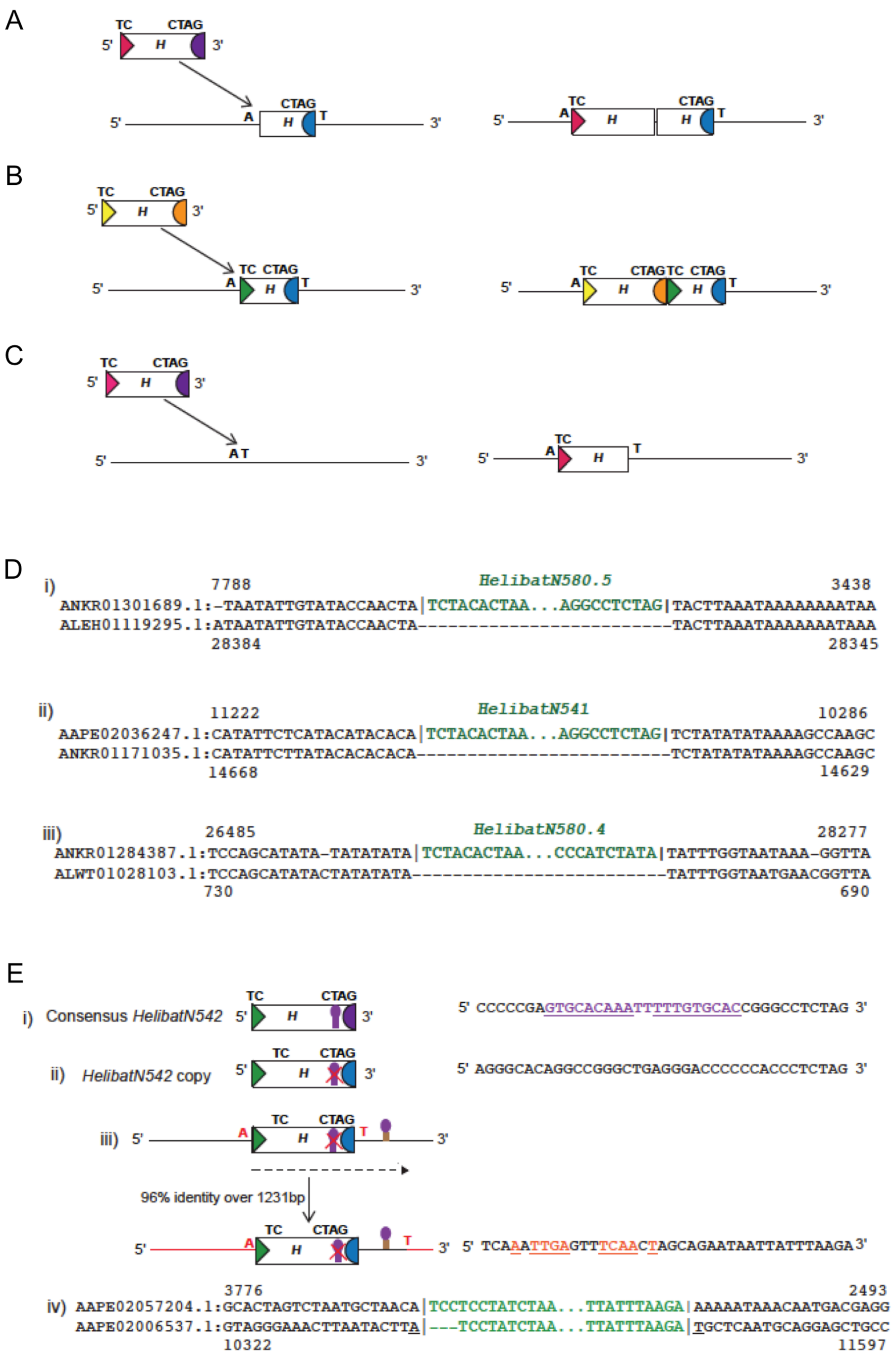

Supplementary Figure 4. Examples of diversification of 3'-ends of *Helitrons* in *Myotis* genomes. **A)** Acquisition of a novel *Helitron* end. Insertion of a *Helitron* copy adjacent to a *Helitron* with truncated 5'-end can lead to acquisition of a novel 3'-end. **B**) Insertion of *Helitron* right next to each other. Insertion of a *Helitron* between 5'-A of the host and T-3' of a *Helitron* can result in insertions, where a 3'-end of one *Helitron* abuts the 5'-end of another *Helitron*. **C)** Generation of a *de novo* end, possibly by the truncation of the 3'-end. **D)** Comparison of host sequences with *Helitron* insertions (described from **A-C**) and the orthologous empty (insertion-free) sites. The first line is the host sequence flanking the *Helitron* insertion. The second line is the orthologous empty site. The sequences in black represent the host sequence and sequence in green represents the *Helitron* sequences. The accession number and coordinates are shown in black. **E)** Generation of *de novo* termini by end-bypass **[i]** The top cartoon shows the structure of the *HelibatN542* consensus. The terminal sequence of the consensus is shown adjacent to the cartoon. The palindrome within the terminus is shown in purple and sequences that comprise the stem of the palindrome are underlined. **[ii]** Cartoon representation of the structure of a *HelibatN542* copy lacking the palindromic sequence, resulting in a different 3'-end. The sequence of the novel 3'-end is shown next to the cartoon. **[iii]** Location of the two *HelibatN542* copies in the genome. The transposition of one copy (shown as black dashed lines) resulted in end-bypass of the CTAG-3' terminus and terminated at a random sequence followed by a short palindrome. The copy was then inserted to a different position (red line) in the genome. The novel terminal sequence including the palindrome is shown next to the cartoon. **[iv]** The first line is the host sequence with the *Helitron* insertion and the novel terminus. The second line is the paralogous copy with the *Helitron* and the novel terminus. The sequences in black represent the flanking host sequence and sequence in green represents the *Helitron* and the captured host sequences. The accession number and coordinates are shown in black.

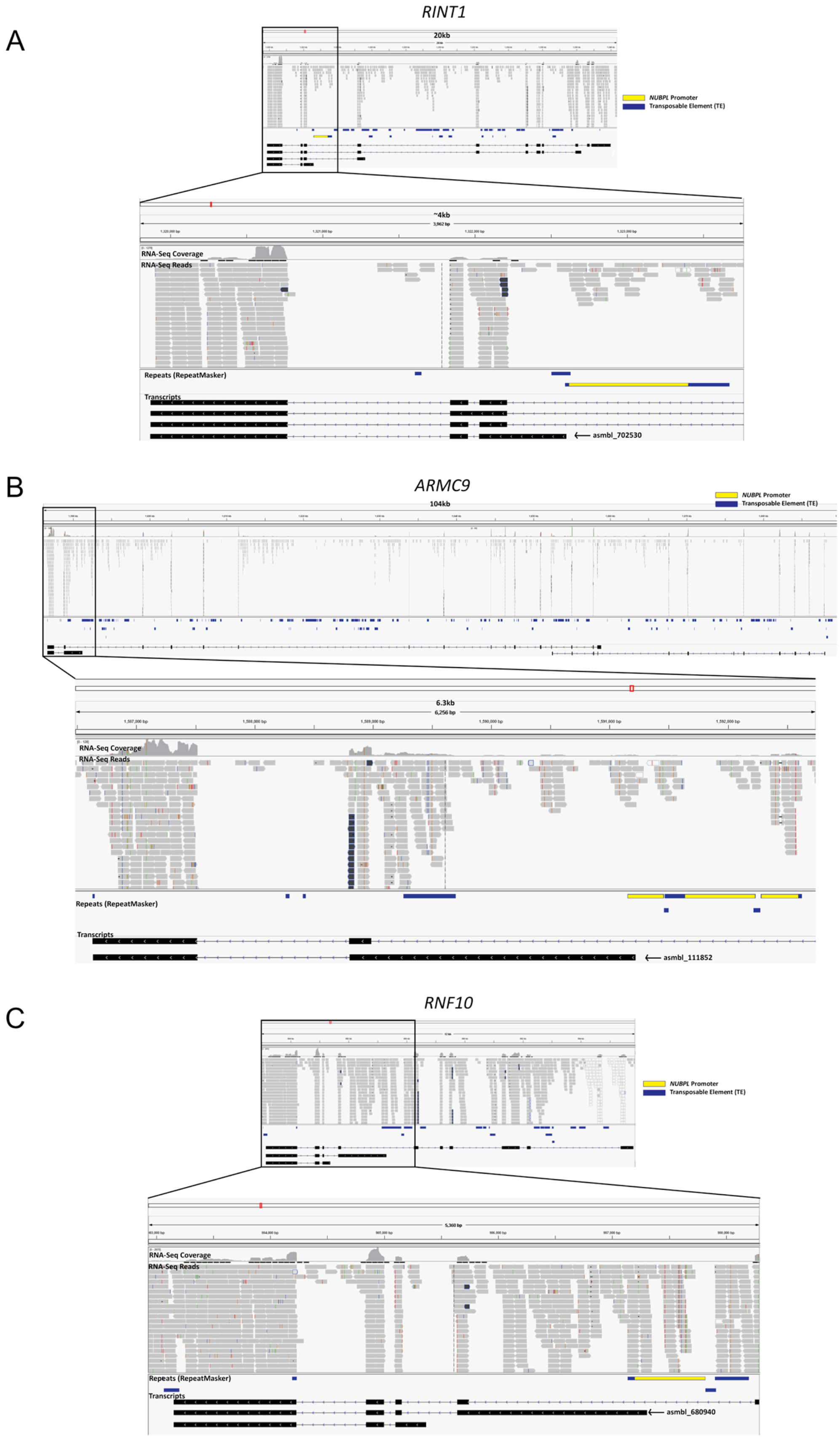

Supplementary Figure 5. *RINT1*, *ARMC9* and *RNF10* loci (*M. brandtii*). A) *RINT1* locus. **B)** *ARMC9* locus. **C)** *RNF10* locus. Shown at the top of each panel is an IGV genome browser snapshot of the full gene models determined by our transcriptome assembly (only transcript assemblies with FPKM >0.5 shown). The expanded versions represent the regions of the gene models that contain the *NUBPL*-driven transcripts. In the expanded versions, the top tracks represent the total coverage of the RNA-seq reads for the gene models, the tracks below show a subset of the reads aligning to the regions. The third tracks indicate the locations of repeats and transposable elements (blue), as well as the locations of the *NUBPL* fragments (yellow). The bottom tracks contain the transcript assemblies (FPKM >0.5), including the transcripts of interest (asmbl_702530 for *RINT1*, asmbl_111852 for *ARMC9* and asmbl_680940 for *RNF10*).

		**In +/- 1 kb TSS**	**Outside +/-1 kb TSS**	***p*-value (Fisher's exact)°**
**All *Helitrons***	**In *Helitrons***	2455[*]	495356[×]	**Left:** $p = 0$<br>**Right:** $p = 1$<br>**Two-tailed:** $p = 0$
**# insertions:** 497811<br>**# genes:** 8829	**Outside *Helitrons***	6374[ǂ]	444254[+]	
		**In +/- 1 kb TSS**	**Outside +/-1 kb TSS**	***p*-value (Fisher's exact)°**
***Helitrons* w/ gene captures**	**In *Helitrons***	643[*]	95878[×]	**Left:** $p = 3.16e\text{-}19$<br>**Right:** $p = 1$<br>**Two-tailed:** $p = 6.017e\text{-}19$
**# insertions:** 96521<br>**# genes:** 8829	**Outside *Helitrons***	8186[ǂ]	861384[+]	

Supplementary Table 1. Contingency Count Table for *Helitron* enrichment analyses in +/-1 kb regions around TSSs in *M. brandtii.*

* # of times *Helitrons* overlap with +/-1 kb regions around TSSs
ǂ # of +/-1 kb regions around TSSs that do not overlap with *Helitrons*
× # of *Helitrons* that do not overlap with +/-1 kb regions around TSSs
\+ # of regions (estimated) that do not overlap with either *Helitrons* or +/-1 kb regions around TSSs
° Left *p*-values indicate probability of *Helitrons* being depleted in +/-1 kb regions around TSSs. Right *p*-values indicate the probability of enrichment, and Two-Tailed the probability of *Helitrons* being different than what is expected by chance.

Name of the *Helitron*	# copies analyzed	# copies with ends similar to consensus	# copies with *de novo* end (different from the consensus end, >20% divergence over the last 30 bps / or do not align)					
			with CTAG termini	End-bypass	Novel *Helitron* end (insertion next to a 5' truncated *Helitron*)	Most likely by deletion	With empty site evidence	No empty site evidence
HelibatN541	26	26	-	-	-	-	-	-
HelibatN542	339	316	4	1	3	5	1	9
HelibatN580	30	13	2	-	2	6	1	6

Supplementary Table 2. Analysis of 3'-ends of recently active *Helitrons* in *Myotis* genomes.

						Promoter		
Transcript	Gene	Coordinates	Scaffold	Expression	FPKM	Origin	Distance from TSS	Approximate Age
asmbl_702530[1]	RINT1	1319873 - 1322604	KE161857.1	Kidney	0.51 (K)	HelibatN3	-1069 to +12	25-12 mya
asmbl_111852[2]	ARMC9	1586631 - 1591216	KE164457.1	Brain	0.65 (B)	HelibatN3	-1020 to +65	40-25 mya
asmbl_680940[1]	RNF10	983155 - 987306	KE161970.1	Constitutive	1.25(B); 1.23 (K); 0.97 (L)	HelibatN3	-894 to +168	40-25 mya
asmbl_43689[2]	RBBP5	1998130 - 2025813	KE164706.1	Constitutive	2.26 (B); 3.47 (K); 0.94 (L)	HelibatN3	-1779 to -764	40-25 mya
asmbl_43690[2]	RBBP5	1998130 - 2025813	KE164706.1	Constitutive	5.22 (B); 2.42 (K); 1.69 (L)	HelibatN3	-1779 to -764	40-25 mya
asmbl_89539[2]	ATG5	9804872 - 9922472	KE164550.1	Constitutive	1.6 (B); 2.3 (K); 4.7 (L)	HelibatN3	-1415 to -614	12-10 mya
asmbl_89540[2]	ATG5	9804872 - 9984397	KE164550.1	Brain/Kidney	1.01 (B); 1.77 (K)	HelibatN3	-1415 to -614	12-10 mya
asmbl_89542[2]	ATG5	9804872 - 9922472	KE164550.1	Brain/Kidney	1.32 (B); 1.45 (K)	HelibatN3	-1415 to -614	12-10 mya
asmbl_89543[2]	ATG5	9804872 - 9964407	KE164550.1	Constitutive	0.91 (B); 0.82 (K); 1.36 (L)	HelibatN3	-1415 to -614	12-10 mya
asmbl_122965[2]	NUP88	599827 - 609108	KE164426.1	Brain	2.34 (B)	HelibatN3	-1527 to -801	40-25 mya
asmbl_133658[1]	PSMB5	513728 - 521120	KE164377.1	Constitutive	19.71 (B); 14.73 (K); 18.87 (L)	HelibatN3	-1467 to -807	40-25 mya
asmbl_406297[1]	ACAP2	5280571 - 5292804	KE163428.1	Kidney/Liver	1.06 (K); 0.91 (L)	HelibatN3	-1390 to -796	12-10 mya
asmbl_406298[1]	ACAP2	5280571 - 5286727	KE163428.1	Brain	2.64 (B)	HelibatN3	-1390 to -796	12-10 mya
asmbl_409885[2]	KIF13A	2335940 - 2346111	KE163411.1	Brain/Kidney	0.64 (B); 0.66 (K)	HelibatN3	-1453 to -796	25-12 mya
asmbl_519813[2]	IL15RA	401446 - 437510	KE162829.1	Liver	0.87 (L)	HelibatN3	-1374 to -668	25-12 mya
asmbl_519842[2]	IL15RA	401446 - 431544	KE162829.1	Liver	0.57 (L)	HelibatN3	-1374 to -668	25-12 mya
asmbl_519905[2]	IL15RA	401446 - 434643	KE162829.1	Liver	0.59 (L)	HelibatN3	-1374 to -668	25-12 mya
asmbl_519907[2]	IL15RA	401446 - 441051	KE162829.1	Liver	0.66 (L)	HelibatN3	-1374 to -668	25-12 mya
asmbl_519908[2]	IL15RA	401446 - 441051	KE162829.1	Kidney	0.53 (K)	HelibatN3	-1374 to -668	25-12 mya
asmbl_519910[2]	IL15RA	401446 - 434643	KE162829.1	Liver	0.86 (L)	HelibatN3	-1374 to -668	25-12 mya
asmbl_519912[2]	IL15RA	401446 - 441051	KE162829.1	Liver	1.93 (L)	HelibatN3	-1374 to -668	25-12 mya
asmbl_519913[2]	IL15RA	401446 - 441051	KE162829.1	Liver	2.96 (L)	HelibatN3	-1374 to -668	25-12 mya
asmbl_519914[2]	IL15RA	401446 - 434643	KE162829.1	Liver	0.64 (L)	HelibatN3	-1374 to -668	25-12 mya
asmbl_541112[1]	ZMYM4	1291579 - 1314976	KE162671.1	Kidney	0.65 (K)	HelibatN3	-1661 to -923	25-12 mya
asmbl_592789[1]	EPS8	3447651 - 3533166	KE162407.1	Kidney/Liver	9.81 (K); 0.75 (L)	HelibatN3	-2025 to -1171	40-25 mya
asmbl_602260[1]	PJA2	5546545 - 5584009	KE162363.1	Brain	2.11 (B)	HelibatN3	-908 to -144	40-25 mya
asmbl_602361[1]	PJA2	5570209 - 5584009	KE162363.1	Liver	0.68 (K)	HelibatN3	-908 to -144	40-25 mya
asmbl_610360[2]	RNF114	3183763 - 3193548	KE162314.1	Brain/Kidney	1.21 (B); 1.06 (K)	HelibatN3	-1718 to -701	40-25 mya
asmbl_244933[2]	ELOVL2	3937750 - 3961655	KE164122.1	Constitutive	7.12 (B); 0.77 (K); 17.52 (L)	HelibatN3	-542 to -135	25-12 mya
asmbl_711145[1]	PROSER1	794021 - 862504	KE161809.1	Brain/Kidney	4.59 (B); 2.1 (K)	HelibatN3	-1456 to -477	25-12 mya
asmbl_710521[1]	FOXJ2	95891 - 114448	KE161817.1	Brain	1.42 (B)	HelibatN264	-888 to -373	< 10 mya
asmbl_710522[1]	FOXJ2	95891 – 114448	KE161817.1	Constitutive	8.01 (B); 5.15 (K); 1.68 (L)	HelibatN264	-888 to -373	< 10 mya
asmbl_547482[2]	CCDC66	1750516 – 1776865	KE162632.1	Constitutive	1.18 (B); 0.84 (K); 0.59 (L)	HelibatN3	-1097 to -95	40-25 mya
asmbl_562141[1]	VPS52	451415 - 452557	KE162539.1	Constitutive	7.53 (B); 5.51 (K); 2.38 (L)	HelibatN3	-1967 to -954	40-25 mya
asmbl_458633[1]	PCKS1	7662370 - 7665976	KE163134.1	Brain	0.61 (B)	HelibatN3	-1982 to -996	40-25 mya
asmbl_280594[2]	HSPH1	3557538 - 3572255	KE163986.1	Constitutive	6.02 (B); 4.97 (K); 1.25 (L)	HelibatN3	-1567 to -530	12-10 mya
asmbl_225943[2]	R3HDM2	275902 - 403344	KE164166.1	Brain/Kidney	3.48 (B); 6.38 (K)	HelibatN3	-2429 to -809	25-12 mya
asmbl_225947[2]	R3HDM2	275902 - 403344	KE164166.1	Brain	0.69 (B)	HelibatN3	-2429 to -809	25-12 mya
asmbl_225950[2]	R3HDM2	275902 - 403344	KE164166.1	Kidney/Liver	1.18 (K); 1.23 (L)	HelibatN3	-2429 to -809	25-12 mya
asmbl_225952[2]	R3HDM2	275902 - 403344	KE164166.1	Brain	5.61 (B)	HelibatN3	-2429 to -809	25-12 mya
asmbl_225955[2]	R3HDM2	275902 - 403344	KE164166.1	Kidney/Liver	1.9 (K); 1.64 (L)	HelibatN3	-2429 to -809	25-12 mya
asmbl_225956[2]	R3HDM2	275902 - 403344	KE164166.1	Brain	1.16 (B)	HelibatN3	-2429 to -809	25-12 mya
asmbl_225958[2]	R3HDM2	275902 - 403344	KE164166.1	Kidney/Liver	1.15 (K); 0.82 (L)	HelibatN3	-2429 to -809	25-12 mya
asmbl_225959[2]	R3HDM2	275902 - 403344	KE164166.1	Kidney/Liver	1.97 (K); 2.48 (L)	HelibatN3	-2429 to -809	25-12 mya
asmbl_226010[2]	R3HDM2	311551 - 403344	KE164166.1	Brain	0.69 (B)	HelibatN3	-2429 to -809	25-12 mya
asmbl_150342[1]	STX10	475164 - 482635	KE164342.1	Brain	0.63 (B)	HelibatN3	-1388 to -357	< 10 mya

[1] NUBPL promoter drives in canonical direction
[2] NUBPL promoter drives in reverse direction
Brain (B); Kidney (K); Liver (L)
Transcripts with TSS provided by *Helitron*-captured promoter

Supplementary Table 3. Candidate *NUBPL*-driven transcripts. This table lists the information for each candidate *NUBPL*-driven transcript including its ID, the name of the gene it belongs to, the scaffold and coordinates of the transcript, and its tissue-specific expression, if any. The information about the specific *NUBPL* promoter insertion is listed on the right side of the table, and includes the donor *Helitron* element, the distance of the element from the TSS (annotated based on our transcriptome assembly; positive number indicates that it overlaps the TSS), and its approximate age, determined as described in Methods. Transcripts labeled in green are those whose TSS is donated by the *NUBPL*-promoter containing insertion. The numbers 1 and 2 indicate the orientation of the transcript. Transcripts denoted with a 1 are driven in the canonical direction by the *NUBPL* promoter, whereas transcripts denoted with a 2 are driven in the reverse direction. Many (11) of these insertions are present in the genomes of the other three sequenced vespertilionid bats (*M. lucifugus*, *M. davidii*, *Eptesicus fuscus*), but there are several (12) lineage-specific insertions, including those in the *FOXJ2* and *STX10* genes that are specific to *M. brandtii*, consistent with *Helibat* activity throughout the diversification of vesper bats[5]. Nine insertions appear to drive their transcripts in the canonical direction, whereas eight insertions drive transcripts in the reverse direction, suggesting that the captured *NUBPL* promoter is bi-directional. This is further supported by the presence of many characteristic promoter sequence features (TATA, CAAT, and GC boxes as well as predicted TF binding sites/overrepresented sites) on both strands of the captured promoter sequence (data not shown). In spite of the small set, these genes are enriched for several GO Terms: protein ubiquitination (GO: 0016567; p=1.295e-02), regulation of signal transduction involved in mitotic G2 DNA damage checkpoint (GO: 1902504; p=1.481e-02), protein modification by small protein conjugation (GO: 0032446; p=1.66e-02), protein modification by small protein conjugation or removal (GO: 0070647; p=3.104e-02), organelle organization (GO: 0006996; p=3.312e-02), cell cycle (GO: 0007049; p=4.082e-02), and actin polymerization-dependent cell motility (GO: 0070358; p=4.439e-02).

Primer name	Primer sequence 5′→3′
Hel_Lft1	GGCGCTTGACACCTGCGTAT
Hel_Lft2	GTGGCTTGAGCGTAGCGGAG
Hel_L_bc	ACACTCTTTCCCTACACGACGCTCTTCCGATCT**_ILLUMINA_TRUSEQ_BARCODE_**TTTG CATGTTTCTCTTTTATTATATAG
Hel_3P_1	ATTAATTCCCTTTCAATGTGCACGAA
Hel_3P_2	TTCCCTTTCAATGTGCACGAATTT
Hel_3P_3BC	ACACTCTTTCCCTACACGACGCTCTTCCGATCT**_ILLUMINA_TRUSEQ_BARCODE_**AATT TCGTGCACCGGGCCACT
Puro1	CCTTCTATGAACGGCTGGGCTT
Puro2	GGGCTTTACTGTGACCGCAGAT
T2a_SD_bc	ACACTCTTTCCCTACACGACGCTCTTCCGATCT**_ILLUMINA_TRUSEQ_BARCODE_**GAAA ACCCTGGACCAATGGTTTGT
PE first	gtgactggagttcagacgtg
PE nest	CAAGCAGAAGACGGCATACGAGAT**_REVERSE_COMPLEMENT_OF_ILLUMINA_TRUSEQ _BARCODE_**GTGACTGGAGTTCAGACGTGTGCTCTTCCGATCT
Illumina 1 (Oligonucleotide sequences ©2006-2010 Illumina, Inc. All rights reserved.	AATGATACGGCGACCACCGAGATCTACACTCTTTCCCTACACGACGCTCTTCCGATCT
Hemispecific primers for 3′ end	GTGACTGGAGTTCAGACGTGTGCTCTTCCGATCTDDDNNNAACG
	GTGACTGGAGTTCAGACGTGTGCTCTTCCGATCTHHHNNNCTAC
	GTGACTGGAGTTCAGACGTGTGCTCTTCCGATCTHHHNNNGGAC
	GTGACTGGAGTTCAGACGTGTGCTCTTCCGATCTNBNNBHCGTT
	GTGACTGGAGTTCAGACGTGTGCTCTTCCGATCTVNVNVNGCAA
	GTGACTGGAGTTCAGACGTGTGCTCTTCCGATCTHNHNNNGTCC
Hemispecific primers for 5′ end	GTGACTGGAGTTCAGACGTGTGCTCTTCCGATCTHHHNHNATTC
	GTGACTGGAGTTCAGACGTGTGCTCTTCCGATCTBBBNNNGAAT
	GTGACTGGAGTTCAGACGTGTGCTCTTCCGATCTHHHNNNGAAC
	GTGACTGGAGTTCAGACGTGTGCTCTTCCGATCTVNVNNNGTAA
	GTGACTGGAGTTCAGACGTGTGCTCTTCCGATCTHNHNNNGTCC
	GTGACTGGAGTTCAGACGTGTGCTCTTCCGATCTHHHNNNTTAC
Hemispecific primers for fusion transcript detection	GTGACTGGAGTTCAGACGTGTGCTCTTCCGATCTNDDNNNAGTG
	GTGACTGGAGTTCAGACGTGTGCTCTTCCGATCTBBBNNNCACT
	GTGACTGGAGTTCAGACGTGTGCTCTTCCGATCTVNVNNNTCAA
	GTGACTGGAGTTCAGACGTGTGCTCTTCCGATCTDVVNNNTTGA
	GTGACTGGAGTTCAGACGTGTGCTCTTCCGATCTHVVNVNTACA
	GTGACTGGAGTTCAGACGTGTGCTCTTCCGATCTSVVNNNTGTA
First Y to Phe*	GCCTCCATCAAATCCGTGAAGTTCCTGTTCAAATACATCTACAAAGGC
Second Y to Phe*	GTGAAGTACCTGTTCAAATTTATCTACAAAGGCCACGACTGC
Double H to Ala*	CAGAAACGGGGCCTGCCAGCAGCCGCAATCCTGCTGATCCTGG
Double H to Gln*	CAGAAACGGGGCCTGCCACAAGCCCAAATCCTGCTGATCCTGG
Double Y to Phe*	GCCTCCATCAAATCCGTGAAGTTCCTGTTCAAATTTATCTACAAAGGC
Helicase K1068Q*	GGACCCGGAGGGTCTGGCCAAACCTACCTGTATAAAGTG
Helicase R1457Q*	CTGTATGTGGCCTTTAGCCAAGTGCGCCGGGCCTGCGAT
HUH NcoI site*	GATAATGTGCCGATTGGCACCATGGTTATTCTGCCGAGCAGTTTTG
HUH stop one*	CAGATTAGCGAAAAAAACTGAATCAACCACGATGAGGTG
First Y to Phe-insect*	GCCAGCATTAAAAGCGTGAAATTCCTGTTCAAATATATCTATAAAGGC
Second Y to Phe-insect*	GTGAAATACCTGTTCAAATTTATCTATAAAGGCCACGATTGC
Double H to Ala-insect*	CAGAAACGGGGCCTGCCAGCAGCCGCAATCCTGCTGATCCTGG
Double H to Gln-insect*	CAGAAACGTGGTCTGCCGCAAGCCCAAATTCTGCTGATTCTGG
Double Y to Phe-insect*	GCCAGCATTAAAAGCGTGAAATTCCTGTTCAAATTTATCTATAAAGGC
Helicase K1068Q-insect*	GGTCCGGGTGGCAGCGGTAACACCTATCTGTATAAAGTG
Helicase R1457Q-insect*	CTGTACGTTGCCTTTAGCAACGTTCGTCGTGCATGTGAT
Helraiser LTS substrate (+)*	CACCATATGATCCTATATAATAAAAGAGAAACATGCAAATTGACCATCCC
Helraiser RTS substrate (+)*	CCCTTTCAATGTGCACGAATTTCGTGCACCGGGCCACTAGTATATATATA
HelMut fwd*	CCCTTTCAATGTGCACGAA CGGGCCACTAGTATATATATAAAGC
HelRDelH*	CTAATTAATTCCCTTTCAAT CGGGCCACTAGTATATATATAAAGC
ATH1	TTATATATATACTAGTGGCCCGACCTGCGGTACACCGCAGGTATTG

ATH2	GCTATTTGCCCTTTCTCTATAATAGAAGTGTGAGAGATGAAAGGAAATGAGTAAAATGTATATGAAAATAATAC
ATH3	GAGAAAGGGCAAATAGCAATATTAAAATATTTCCTCTAATTAATTCCCTTTCAATACCTGCGGTGTACCGC
ATH4	TATCATGTCTGGATCCAAATTTATGTATTATTTTCATATAC
ATH5	TTATATATATACTAGTGG
ATH6	TATCATGTCTGGATCC
LX1	TTATATATATACTAGTGGCCCGGTGCACGACGGACGTGCACATTG
LX2	GCTATTTGCCCTTTCTCTATAATAGAAGTGTGAGAGATGAAAGGAAATGAGTAAAATGTATATGAAAATAATAC
LX3	GAGAAAGGGCAAATAGCAATATTAAAATATTTCCTCTAATTAATTCCCTTTCAATGTGCACGACGGACGTGCACCGGGCC
LX4	TATCATGTCTGGATCCAAATTTATGTATTATTTTCATATAC
LX5	TTATATATATACTAGTGG
LX6	TATCATGTCTGGATCC
SX fwd	AATTTCCGCAGGTCGGGCCAC
SX rev	CCGCAGGTATTGAAAGGG
Hel1	CCTCCTGGGGCGCTTGACACCTGCG
Hel2	TGGCTGGTGGGCGTGGCTTG
Hel5	TCATCTCTCACACTTCTATTATAGAG
Linker primer	GTAATACGACTCACTATAGGGC
Nested primer	AGGGCTCCGCTTAAGGGAC
HelCD1	GGCAGTTAAATTTGCATACGCAG
WT6a	CAGTTACCTAGAAGGAAACAGAG
WT6b	GTCACAGCCCATGATATGCCC
WT6c	CTTGCTGTTTGAATATGAAATTATGTTATTC
WT6d	CATTATGCCAATTTCACAGATGAGG
DelH2	GAAGGTAATTTAGAAGTGAAAGAACAC
DelH14	GTATCTATCACCTCACCTAGTTAAC
DelH19	GCTGGAACGTTAATTATGATGCG
DelRTS2	GTTGATATGGAAGATGAGAATGAAAC
DelRTS15a	CTGACAGGATTTTGGAGAATACG
HA tag top	gactctactagtgccaccATGTACCCTTACGACGTACCGGATTACGCC TACCCTTACGACGTACCGGATTACGCCactagtgactct
HA tag bottom	agagtcactagtGGCGTAATCCGGTACGTCGTAAGGGTAGGCGTAATC CGGTACGTCGTAAGGGTACATggtggcactagtagagtc
Universal primer for the ssDNA LM-PCR	CGCTGGAAGCTTAAG
5+ primer for the ssDNA LM-PCR	GCGCGGGAATTCCACCATATG
5- primer for the ssDNA LM-PCR	GCGCGGGAATTCGGGATGGTCAATTTGC
3+ primer for the ssDNA LM-PCR	GCGCGGGAATTCCCCTTTCAATGTGCACG
3- primer for the ssDNA LM-PCR	GCGCGGGAATTCTATATATATA

Supplementary Table 4. List of primers. For primer pairs where reverse primer is reverse complement of the forward primer sequence, only forward primer sequence is shown.

Supplementary Notes

Supplementary Note 1

Molecular reconstruction of the *Helraiser* transposon

Using a set of ~300-aa protein sequences corresponding to the conserved rolling-circle replication initiator domain (Rep) present in the RepHel proteins encoded by diverse known *Helitrons* in plants and metazoans, we identified all bat sequences coding for this domain by using them as queries in a Censor[6] search against the GenBank *Myotis lucifugus* assembly. To check if the identified DNA sequences might have been composed from different families, we performed their clustering by BLASTCLUST (standalone Blast, NCBI). Based on the clustering results, we concluded that the bat genome contained only one major family of autonomous-like *Helitrons*. All these sequences, even those contaminated by premature stop codons and short indels, have been used to derive a ~900-bp Rep consensus sequence coding for the catalytic domain. At the next step, genomic sequences >90% identical to the 200-bp 5'- and 3'-terminal parts of the Rep consensus have been expanded up to 2 kb upstream and downstream of the termini, respectively. For the two sets of multiple alignments of the expanded sequences two consensus sequences were derived. These two terminal consensuses and the Rep consensus have been assembled together into one long expanded consensus. This procedure has been iteratively repeated till both ends of the bat autonomous-like *Helitron* have been identified, and the first version of the autonomous bat consensus sequence (*Helitron-1_ML*) was built.

Next, by using Censor, we collected all copies in the *M. lucifugus* genome >90% identical to the *Helitron-1_ML*. Based on the pairwise alignment of the collected sequences expanded 1 kb in both directions, we removed all copies that were generated by long segmental duplications (>90 % identical to each other) unrelated directly to the multiplication of *Helitrons* by their transposition. As a result we collected a final set composed of 500 copies of *Helitron-1_ML.* After multiple alignment of all these sequences and *Helitron-1_ML*, we derived a second version of the consensus, a 5301-bp *Helitron-1a_ML* coding for a 1458-aa RepHel protein and ~95 % identical to the collected 500 copies.

The consecutive analysis of the *Helitron-1a_ML* copies revealed that the genome contains only a small number of autonomous-like copies, when the majority of the copies are in fact copies of two non-autonomous *Helitron-1N1_ML and Helitron-2N2_ML* transposons*.* The 2437-bp *Helitron-1N1_ML* and 2144-bp *Helitron-1N2_ML* consensus sequences encoded the 610-aa N-terminal and 390-aa C-terminal portions of the *Helitron-1a_ML* RepHel protein, respectively. Presumably, these non-autonomous transposons were transposed by the RepHel transposase expressed by some autonomous *Helitrons*. Therefore, we concluded that the regions in the non-autonomous transposons coding for remnants of RepHel may contain mutations that can destroy or damage proper functions of the protein reconstructed from copies of the non-autonomous elements. To avoid this problem, copies of the non-autonomous transposons were excluded from the "500-fragments" set. As a result, only 46 sequences, supposedly fragments of the real autonomous *Helitron*, have remained in the modified set. Based on re-alignment of the *Helitron-1a_ML* consensus sequence with these 46 sequences, a new 5295-bp *Helitron-1b_ML* consensus sequence encoding the 1494-aa RepHel protein was derived (~95 % identity between the consensus and 46 sequences).

At this point *Helitron-1b_ML* and *Helitron-1a_ML* consensus sequences were 98.81 % identical and the RepHel proteins encoded by these consensus sequences differed from each other by 13 aa replacements and by a 36-aa C-tail added to the *Helitron-1b_ML* coding sequence.

Since the sequences in the original "500-fragments" set have not contained short fragment of long autonomous *Helitrons* generated by insertions of other transposable elements and by internal deletions, for each of the 46 coding sequences the *Helitron-1b_ML* was derived from, all terminal and additional internal fragments were manually added creating thereby a set of 177 fragments. Based on re-alignment of the *Helitron-1b_ML* consensus with all these fragments, the final version of the 5296-bp autonomous consensus sequence that we named *Helraiser* was derived (**Supplementary Fig. 1**).

Supplementary Note 2

Constructs

Transposase vectors. The coding region of the *Helraiser* transposase was synthesized by GenScript following human codon optimization, and cloned by *Spe*I/*Xho*I into the expression vector FV4a[7] to yield the transposase helper plasmid pFHelR. An N-terminal 2XHA-tag was inserted as a synthetic double-stranded oligonucleotide encoding MYPYDVPDYAYPYDVPDYA into the *Spe*I site of pFHelR to yield pF-HA-HelR. The CMV promoter-driven transposase expression plasmid pCHelR was generated by inserting the *Spe*I/*Xho*I fragment of pFHelR into the *Nhe*I/*Xho*I sites of pcDNA3.1(-) (Invitrogen). To create the pCHelRGFP plasmid, the *Xho*I/*Not*I fragment of pMSCV20Ires-GFP (from B. Schroeder, MDC) was inserted into the *Xho*I/*Not*I sites of pCHelR. Transposase catalytic mutant expression plasmids were generated by mutagenic PCR using pCHelR as a template. The transposase vector used for *Helraiser* protein expression in insect cells was generated by subcloning the *Helraiser* transposase coding sequence synthesized by GENEART (Invitrogen) into pFastBac HT-A (Invitrogen) using *Nco*I and *Xho*I restriction sites.

Transposon vectors. An SV40-puro or SV40-neo selection cassette was cloned between the consensus LTS and RTS sequences of *Helibat1* (pHelR), *HelibatN1*, *HelibatN2* and *HelibatN3* that were synthesized by GeneScript (**Supplementary Fig. 1**). Transposon donor vectors pHelRΔHP, pHelRMut, pHelRATH, pHelRStemX and pHelRLoopX were generated by deletion or replacement of the palindromic sequence in the transposon 3'-end. pHelRMut and pHelRΔHP vectors were created by deletion PCR using primer pairs: Hel-Mut fwd/Hel-Mut rev, and HelRDelH fwd and HelRDelH rev, respectively. To generate pHelRATH and pHelRLoopX donor plasmids four oligonucleotides ATH1, ATH2, ATH3, ATH4 and LX1, LX2, LX3, LX4, respectively, were annealed in equimolar ratios (0.8 μM each oligo, 0.2 mM dNTP mix and 1 μl PfuUltra II Fusion HotStart DNA Polymerase (Agilent technologies) / 50 μl reaction). The temperature profile for the oligo annealing reaction was 10 cycles at 95 °C for 20 s, 72 °C for 10 s. 1 μl of the annealing reaction was used for the PCR amplification of the ATH or LX fragments using the ATH5/ATH6 and LX5/LX6 primer pairs, respectively. In the final step, ATH and LX PCR fragments were digested by *Spe*I and *BamH*I and cloned into the *Spe*I/*Bam*HI sites of pHelR. To generate the pHelRStemX transposon donor plasmid, pHelRATH was used as a template in mutagenesis PCR together with the primers SX fwd and SX rev. After the PCR reaction the ends of the linear fragment were ligated together, thereby generating pHelRStemX. To create pHelRΔRTS,

pHelR was digested with *Spe*I/*Bam*HI restriction enzymes. The restriction sites were blunted with Klenow (Fermentas) and re-ligated. The pHelRΔLTS donor plasmid was generated through *Nde*I and *Eco*RI digestion of the Hel1C backbone followed by Klenow treatment of the restriction sites and vector backbone re-ligation. The pHelRPN and pHelRΔHPN donor plasmids were generated by inserting the *Spe*I fragment from the pUC19SBneo[8] vector into the *Spe*I site of the pHelR and pHelRΔHP vectors, respectively. To generate the *Helitron* circle donor plasmid pHelRCD, first pIRES-EGFP-N1 vector was constructed by cloning the *Not*I/*Bam*HI fragment of pWAS-EGFP into the *Not*I/*Bam*HI sites of the pGFP-N1 plasmid (Clontech). The *Eco*RI/*Bam*HI fragment of the pIRES-EGFP-N1 plasmid was then cloned into the *Eco*RI/*Bam*HI sites of the pHelR plasmid, thereby creating pHelRCD. The pHelRCneo vector was created by inserting the *Bam*HI/*Eco*RI fragment from pHC plasmid (generated through *Helraiser* transposition from the pHelRCD donor plasmid in HeLa cells) into the *Bam*HI/*Mfe*I sites of pcDNA3.1(-). In the next step, the *neo* coding sequence in pHelRCneo was exchanged with the *puro* coding sequence from the pHel1C plasmid using the *Avr*II/*Bam*HI restriction sites, thereby generating the pHelRCpuro vector. The *Helitron* circle vector with the deletion of the palindromic sequence in the transposon 3'-terminus, pHelRCΔHPpuro, was generated *via* site directed mutagenesis PCR using pHelRCpuro as a template and Hel-Mut fwd/Hel-Mut rev primer pair. The integrity of all coding regions and transposon constructs generated by PCR was verified by DNA sequencing.

Supplementary Note 3

Circle detection assay

Helraiser circle formation in HeLa cells was confirmed by circle detection PCR. First, 2×10^5 HeLa cells were seeded onto six well plates one day prior to transfection. 48 hours post-transfection, plasmids were isolated from the cells using a modified Qiagen QIAprep Spin Miniprep protocol using 300 µl 1.2 % SDS supplemented with 50 µg of Proteinase K in the cell lysis step instead of the P2 buffer. The rest of the plasmid isolation procedure was performed according to the manufacturer's protocol. 150 ng of isolated plasmid was used for PCR with the primers Hel1 and Hel5. The temperature profile for PCR was: 98 °C for 2 min, followed by 34 cycles of 98 °C for 10 s, 59 °C for 15 s, 72 °C for 10 s. The final elongation was performed at 72 °C for 5 min.

Supplementary Note 4

***Helraiser* re-transposition in HeLa cells**

For re-transposition assays, H1 cells were grown on a 100-mm plate (2 µg/ml puromycin) until confluency. One day prior to transfection, 2×10^6 cells were seeded onto a new 100-mm plate. Twenty µl of jetPRIME transfection reagent and 500 µl of jetPRIME buffer were used to transfect 3.5 µg of pCHelRGFP plasmid to the cells. Forty-eight hours after transfection, cells were FACS-sorted for GFP expression and 5×10^5 GFP-positive cells were plated on a 150-mm plate (2 µg/ml puromycin) and left to grow for one week. The procedure was repeated two more times with seven days between the cycles, each time using those cells for transfection that were FACS-sorted the week before. After the cells were transfected and FACS-sorted for the third time, they were grown on a 150-mm plate (2 µg/ml puromycin) until confluency and pooled for genomic DNA isolation and insertion site analysis.

Supplementary Note 5

Insertion site- and copy number analysis by splinkerette PCR

HeLa cell clones were grown until confluency on 6-well plates, washed with PBS and incubated overnight at 55 °C with shaking in lysis buffer (100 mM Tris pH 8.0, 5 mM EDTA, 0.2 % SDS, 200 mM NaCl and 100 µg/µl proteinase K). HeLa genomic DNA (gDNA) was isolated from lysed cells with standard phenol/chloroform extraction. Five µg of gDNA was digested with *Fsp*BI for four hours followed by ethanol precipitation. In the next step, samples were ligated (300 ng) to *Bfa*I splinkerette adapters (100 pmol) in 20 µl reactions. Three microliters of the ligation reaction were used for the first PCR with primers Linker primer and Hel1. The temperature profile for the first PCR round was: one cycle of 94 °C for 3 min, followed by 15 cycles of 94 °C for 30 s, 70 °C for 30 s and 72 °C for 30 s; 5 cycles of 94 °C for 30 s, 63 °C for 30 s and 72 °C for 2 s with an increase of 2 s per cycle; 5 cycles of 94 °C for 30 s, 62 °C for 30 s and 72 °C for 12 s with an increase of 2 s per cycle; 5 cycles of 94 °C for 30 s, 61 °C for 30 s and 72 °C for 22 s with an increase of 2 s per cycle and 5 cycles of 94 °C for 30 s, 60 °C for 30 s and 72 °C for 30 s. Nested PCR was performed with primers Nested and Hel2, and 1 µl of a 1:100 dilution of the first PCR was used per 50 µl reaction. The temperature profile for the nested PCR started with a cycle of 3 min at 94 °C followed by 10 cycles of 94 °C for 30 s, 65 °C for 30 s and 72 °C for 30 s and 20 cycles of 94 °C for 30 s, 55 °C for 30 s and 72 °C for 2 min. The final elongation was performed for 5 min at 72 °C.

In order to analyze transposon-genome junction sites at the 3'-terminus of the *Helraiser* insertions generated with the pHelR, pHelRΔHP and pHelRΔRTS transposons, first left-end splinkerette PCR was performed with the gDNA isolated from HeLa clones to determine genomic locations of the transposon insertions. In the next step, specific primers complementary to the genomic sequence located between 50 and 100 bp downstream from each transposon insertion were designed (WT6a, WT6b, WT6c, WT6d, DelH2, DelH14, DelH19, DelRTS2, DelRTS15a), and used in genomic PCR together with the HelCD1 primer complementary to the sequence at the 5'-terminus of the *Helraiser* transposon. The temperature profile for PCR was: 95 °C 2 min, followed by 40 cycles of 95 °C 20 s, 57 °C 20 s, 72 °C 90 s. The final elongation step was performed at 72 °C 5 min. PCR products obtained in the genomic PCR were sequenced and analyzed.

Supplementary Note 6

Protein expression and purification

Cell pellets were resuspended in Nickel affinity column binding buffer (20 mM NaH_2PO_4 pH 7.4, 500 mM NaCl, 50 mM imidazole, 1 mM TCEP). All subsequent steps were performed at 4 °C. Lysis was done by incubating the cells on ice for 30 minutes, then sonication with a Misonix Sonicator 3000 (5x 20-sec pulses with 3 minute pause at 82 Watts). The soluble fraction was isolated by centrifugation at 20,000 x *g*, loaded onto a HiTrap CHeLating column (GE Healthcare) equilibrated in Nickel affinity column binding buffer, and eluted using a linear gradient with elution buffer (20 mM NaH_2PO_4 pH 7.4, 500 mM NaCl, 250 mM imidazole, 1 mM TCEP). The eluted protein was dialyzed overnight in 20 mM NaH_2PO_4 pH 7.0, 250 mM NaCl, 1 mM DTT and 1mg/ml TEV protease added at 1:100 protease to protein volume ratio. The product was loaded onto a HiTrap Heparin HP column (GE Healthcare) pre-equilibrated with Heparin column binding buffer (20 mM NaH_2PO_4 pH 7.0, 250 mM NaCl, 1 mM TCEP), and eluted using a linear gradient with elution buffer (20 mM NaH_2PO_4 pH 7.0, 2 M NaCl, 1 mM TCEP). The *Helraiser* transposase was loaded on a HiLoad 16/60 Superdex 200 sizing column (GE Healthcare) equilibrated

with 50 mM HEPES pH 7.5, 150 mM NaCl, 0.5 mM EDTA, 1 mM TCEP, and fractions containing the purified protein were concentrated to 10 mg/ml. All point mutants were purified in the same manner, and exhibited no changes in either expression or purification behavior (>90 % homogeneity) from that of the wild-type transposase. The same procedure was also used to purify truncated versions of the transposase.

Supplementary Note 7

Cleavage assay and sequencing of cleavage products

Cleavage was done at 37 °C for 1 hr, and quenched by addition of 2 µl Proteinase K (New England BioLabs) and 2 µl of 0.5 M EDTA. For reactions with 5 mM $MgCl_2$, reaction was done overnight at 37 °C. Proteinase K digestion was at 45 °C for 30 min, after which an equal volume of loading dye (80 % formamide, 1 mg/ml xylene cyanol, 1 mg/ml bromophenol blue, 10 mM EDTA) was added and reactions incubated at 22 °C for 15 min, and then five min at 95 °C prior to gel loading on 15 % Tris/Borate/EDTA/Urea gels (Invitrogen). The results were visualized using a Typhoon Trio (GE Healthcare).

Gels were stained with SYBR Safe DNA gel stain (Invitrogen), visualized by blue light, and each band cut out. ssDNA extraction was done by crushing the gel and shaking overnight at 37 °C in Extraction Buffer (0.5 N NH4Ac, 10 mM MgAc, 1 mM EDTA, 0.1% SDS). To remove any remaining contaminants, the solution was centrifuged at 14,000 x *g* for 2 min at 4 °C, and the supernatant further cleaned of salts using an Illustra MicroSpin G-25 Columns (GE Healthcare). ssDNA was ligated using ssDNA ligase kit (New England Biolabs) to the following oligonucleotide: 5'-/5rApp/CAAGGATCTTACCGCTGTTGAGATCCAGTTCGATGTAACCCACTCGTGCACCCAACTGATCTTCAGCATCTTTTACTTAAGCTTCCAGCG/3SpC3/-3'. Then using PCR and primers designed for the known part of the sequence of the 5' end and reverse primer to the above oligonucleotide, the fragment was amplified. The resulting dsDNA was cloned into pUC19 using *Eco*RI and *Hin*dIII restriction sites, and sequenced at the FDA-FBR facility.

Supplementary Note 8

Protease digest and N-terminal sequencing

Helraiser transposase was diluted at 1 mg/ml in 20 µl of digestion buffer (50 mM Hepes pH 7.5, 150 mM NaCl, 5 mM $MgCl_2$, 1 mM TCEP), and a series of trypsin dilutions were added to final concentrations ranging from 0.1-1 µg/µl. Samples were incubated at 37 °C for 1 hr, and reactions quenched with NuPAGE loading dye (Novex) and boiling at 95 °C for 5 min. Samples were then immediately loaded onto a 4-12 % NuPAGE bis Tris gel (Novex). Bands were transferred to blot paper using Invitrogen's iBlot kit, and the sequence of each N-terminal sequence was determined by the FDA-FBR facility.

Supplementary Note 9

Colorimetric ATPase assay

ATP hydrolysis was analyzed by measuring the formation of free phosphate (Pi) as a function of time using procedures adapted from Baykov *et al.*[9]. *Helraiser* transposase or mutant proteins were diluted to final concentrations between 0.3-1 µM in buffer containing 50 mM HEPES pH 7.5, 100 mM NaCl, 1 mM DTT and 2 mM $MgCl_2$ and then heated to 37 °C for 10 min. Reactions were initiated by the addition of ATP (Jena biosciences) to either a final concentration of 1 mM or a

concentration range between 0.0078 and 1 mM in a total volume of 180 µl. Samples (20 µl) were removed at various time points and immediately quenched in wells of a 96-well plate, each containing 5 µl of 0.5 M EDTA. An aliquot (150 µl) of a 1 mM malachite green stock solution was added to each well, and the absorbance at 650 nm was measured using a Molecular Devices Spectramax M5 microplate reader. The amount of phosphate released was calculated by comparison to a standard curve generated using KH_2PO_4. DNA stimulation of ATP hydrolysis was measured using the same buffer and protein concentration range (0.3, 0.08, 0.02 mM), and ATP (1 mM), but with the addition of 1 µM of either a 50-base-long ssDNA or 50-bp-long dsDNA prior to the addition of ATP. Calculations of K_m and k_{cat} were done in EXCEL (Microsoft) and KaleidaGraph 4.0.

Supplementary Note 10

Integration site and fusion-transcript library construction

The generation of the insertion site and fusion-transcript libraries was based on a computation-assisted hemi-specific PCR scheme. The PCR assays relied on the use of hemi-specific primers[10] carrying only 4 specific nucleotides (4-mers) at their 3'-ends followed by random sequences and a specific overhang. These primers are to anneal to the neighborhood of the transposon-genome or transposon–genomic transcript junctions of the template genomic DNA, or cDNA, respectively, in order to tag these loci for nested PCR amplifications. The 4-mers of the hemi-specific primers were designed computationally. Possible 4-mers were ranked by their representation in the human genome or transcriptome, excluding those which could give rise to unwanted amplicons on the transposon sequences or on primer overhangs. Similarly, an algorithm was implemented to predict the combination of those six 4-mers, which result in the most comprehensive library for the human genome, or transcriptome, and the transposon vectors used. Next, multi-step PCR schemes were performed to obtain indexed, Illumina-flow cell compatible fusion transcriptome, or integrome libraries.

Supplementary Note 11

Insertion-library preparation and high-throughput sequencing of integration sites of *Helitron* transposons in the human genome

300 ng of gDNA isolated from pools of puromycin-resistant HeLa colonies were used as template for the initial 6 parallel PCR reactions, containing 6 different hemi-specific primers, with the following conditions: for the 5' *Helitron* transposon end: 95 ^{o}C 1 min, 40 cycles of (94 ^{o}C 30 s, 65 ^{o}C 30 s, 72 ^{o}C 30 s), 2 cycles of (94 ^{o}C 30 s, 25 ^{o}C 1 min, ramp to 72 ^{o}C at 0.2 ^{o}C/s, 72 ^{o}C 1 min) with 5 pmol of Hel_Lft_1 specific for the 5'-*Helitron* sequence or 5 pmol of Hel_3P_1 for the 3'-transposon end with the same program but with 62 ^{o}C annealing temperature. The first PCR reactions were supplemented with 25 µls of PCR master mix containing 15 pmol of Hel_Lft_2 for the 5'- and Hel_3P_2 for the 3'-transposon end, respectively. The PCR program for the 5'-end was: 15 super-cycles of [3 cycles of (94 ^{o}C 30 s, 65 ^{o}C 30 s, 72 ^{o}C 40 s) 1 cycle of (94 ^{o}C 30 s, 60 ^{o}C 30 s, 72 ^{o}C 40 s)]. For the 3'-end 62 ^{o}C annealing temperature was used for the 3 cycles. The PCR products were column-purified and 2 µls of the 30 µl elutes were used for the 1st exponential PCRs, with the primer PE_first and Hel_L_bc for the 5'- and Hel_3P_bc for the 3'-transposon ends, respectively, using the following cycling conditions: 95 ^{o}C 30 s, 20 cycles of 94 ^{o}C 30 s, 65 ^{o}C 30 s, 72 ^{o}C 1 min. For the 3'-transposon end the annealing temperature was 58 ^{o}C. 1 µl 10x diluted 1st exponential PCR

products were used to add Illumina adaptors to the amplicons using Pfx polymerase (Life Technologies) with these cycling conditions: 95 °C 30 s, 20 cycles of 94 °C 15 s, 68 °C 1 min. The final PCR products were run on agarose gels and amplicons between 200 and 500 bp were excised and column-purified (Zymoclean Gel DNA Recovery Kit, Zymo Research). The sequencing of the resulting libraries was carried out on Illumina HiSeq 2500 instruments at the Beckman Coulter Genomics Danvers Massachusetts USA sequencing facility.

The raw reads were processed for mapping as follows. Primer-, transposon-, and right Illumina adapter-related sequences were trimmed. The resulting reads were quality filtered by omitting reads containing 'N' bases and by trimming reads as soon as 2 of 5 bases has quality encoding less than phred score 20. All trimmed reads shorter than 24 bases were dropped. The remaining sequences were mapped against the h19 human genome assembly with Bowtie[11].

Supplementary Note 12

Detection of *NUBPL* promoter-driven fusion transcripts

500 ng of total RNA purified from puro resistant HeLa colonies were reverse-transcribed using Maxima Reverse Transcriptase (Thermo Scientific) and oligo dT primers at 50 °C for 30 min. After heat-inactivation the reverse-transcription reaction was repeated. The RNA was hydrolyzed with one-fifth volume of 1 N NaOH and 0.5 M EDTA at 65 °C for 15 min. The cDNA was purified with DNA Clean & Concentrator Kit (Zymo Research) and 2 µl of the elute was used for 6 independent PCR amplifications with the following conditions: 95 °C 1 min, 40 cycles of (94 °C 30 s, 65 °C 30 s, 72 °C 30 s), 2 cycles of (94 °C 30 s, 25 °C 1 min, ramp to 72 °C at 0.2 °C/s, 72 °C 1 min) with the primer Puro1 specific for the *Helitron* vector sequence and 4-mer hemi-specific primers computationally predicted for high representation on the entire human transcriptome. The first PCR reaction was supplemented with 25 µls of PCR master mix containing the vector specific oligo Puro2 to perform the subsequent asymmetric PCR reaction with the following condition: 10 super-cycles of [3 cycles of (94 °C 30 s, 65 °C 30 s, 72 °C 40 s) 1 cycle of (94 °C 30 s, 60 °C 30 s, 72 °C 40 s)]. The PCR products were column-purified and 2 µl of the 30 µl elute were used for the 1st exponential PCR, with the transposon-specific oligo T2a_SD_bc and PE_first specific for the overhang of the hemi-specific primers. The PCR products were purified and TA-cloned using the pGEM-T Vector System (Promega) and sequenced. Fusion transcripts were determined by aligning the sequences following the splice donor site within the *Helitron* transposon with the BLAT tool of the UCSC genome browser.

Supplementary Note 13

***Helraiser* insertion site analysis**

We identified 1751 independent integration events. For statistical analysis, we created sets of randomly chosen genomic sites according to two different background models. The model ('random') is normalized relative the abnormal karyotype of HeLa cells. The second model ('control') also accounts for the mappability of sequencing reads and imitates the base composition at integration sites. To determine the karyotype of HeLa cells, we used ChIP-Seq input data sets generated by the Broad/MGH ENCODE group. Since these data sets were generated without the application of specifically binding antibodies, the read densities can be used as estimates for the relative copy numbers of the underlying genomic regions. Mapped sequencing reads of two biological replicates for HeLa cells as well as for 12 other cell types with normal karyotype

were downloaded from the UCSC Genome Bioinformatics web site (http://genome.ucsc.edu/cgi-bin/hgFileUi?db=hg19&g=wgEncodeBroadHistone). We computed for each data set pair of HeLa cells and normal cells the fold changes of the read counts in sliding windows each covering 1000 consecutive reads from the normal cell data set. The resulting fold changes were multiplied by the assumed average ploidy of HeLa cells (i.e. 3) and divided by ploidy of the normal cells (i.e. 2 for non-sex chromosomes, and chrX and chrY depending on the gender of the control cell data set), then smoothed with a running median filter of window size 30000, and finally rounded to the closest integer value. The results from all pairs of HeLa cell and normal cell data sets were then joined by computing medians. Tested on data originating from normal cells, the method correctly predicted normal karyotypes (data not shown). For the 'random' background model we sampled 500000 random positions in the genome in a way that the probability for choosing a genomic position is proportional to the ploidy of its chromosomal fragment. The 'control' background model was generated as follows. First we sampled 100 million random genomic positions in a way that the probability for choosing a genomic position is proportional to the ploidy of its chromosomal fragment. From these positions we sampled mock sequencing reads having the same length distribution as the real sequencing reads mapped at transposon integration sites. The mock reads were then processed as the sequencing reads described before. The resulting mock sites were scored using a position specific weight matrix (PWM) derived from base composition at integration sites (**Fig. 5A**). From the mock sites we sampled 100,000 control sites in a way that their PWM score distribution resembled the PWM score distribution of the real integration sites. The information about gene expression levels, histone modifications and chromatin accessibility, and the genomic locations of CpG islands and lamina associated domains was downloaded from UCSC (http://genome.ucsc.edu). Open chromatin regions are derived from DNaseI HS data, FAIRE data and ChIP data, validated regions taken from the UCSC Open Chrom Synth track, release 2 (Feb 2012).

Supplementary Note 14

Analysis of 3'-ends of recently active *Helitrons* in *Myotis* genomes

To understand the pattern of acquisition of *de novo* ends by *Helitrons* in sequenced genomes, we analyzed the copies of three *Helibat* exemplars (*HelibatN541*, *HelibatN542* and *HelibatN580*) in the *Myotis* lineage. The copies were recently active (98-99 % identical to the consensus), which minimizes the impact of selection on how the sequence signature is interpreted. The *HelibatN541* copies are unique to the *M. lucifugus* lineage[5], the *HelibatN580* copies unique to the *M. brandtii* lineage and the *HelibatN542* copies are found in both lineages. Copies of *Helibat* exemplars (*HelibatN541* and *HelibatN580*) that were 98-99 % identical to the consensus were extracted from their respective genomes. The *HelibatN542* copies that were >95 % identical to the consensus and have intact 5'-ends were extracted from the *M. lucifugus* genome. Since the *HelibatN542* copies are comparatively older, we used a different cut off. The last 30 bp of each copy were aligned to their respective consensus using MUSCLE[12]. The copies that have ends that are >20 % diverged from the consensus or that do not align (*de novo*) were carefully analyzed using homology-based tools (BLAST tools[13]) to gain insights into the origin and evolution of the 3'-end. We also employed a comparative genomics approach using other bat whole genome sequences to exclude false positives. For example, if a copy in one bat genome has a *de novo* 3'-end and the orthologous copy has an end

homologous to the consensus, then those changes were presumed to have occurred post insertion. In addition, empty sites (insertion-free sites) were used to confirm the boundaries of the element.

Supplementary Note 15

Annotation of *Helitrons* (coordinates, approximate age, and relative orientation) in the *M. brandtii* genome

Helitron insertions were identified (RepeatMasker v.4.0.5 http://www.repeatmasker.org) in the *M. brandtii* genome assembly (KE161034-KE332376, 171343 scaffolds from GenBank at National Center for Biotechnology Information (NCBI)[14] using a *Myotis*-specific *Helitron* repeat library previously described[5]. Conservation of the *Helitron* insertions were determined by taking the *Helitron* DNA sequence plus 200 bp flanking sequence and performing a blastn query of the NCBI wgs database to determine if the insertion was present in other sequenced *Vespertilionidae* bats (*E. fuscus*, *M. lucifugus*, and *M. davidii*). If there was a hit to the entire length of the query sequence in a given species, it was considered present (conserved) in that species. If there was only a hit to the *Helitron* or no hit, it was considered absent. By combining this information with the known divergence times of the bats, we obtained an approximate age for each insertion. To determine if there was a bias in the orientation of *Helitrons* inserting into gene models, we used a pipeline previously described[15] to identify *Helitrons* either overlapping with introns, exons, or in regions 1 kb up or downstream of an annotated gene model. Both *Helitrons* that inserted in the same orientation and opposite orientation as their target gene were quantified, and compared using a 2-tailed, 2-sample T-test, $\alpha = 0.05$.

Supplementary Note 16

***M. brandtii* transcriptome assembly, alternative splicing analysis, abundance estimation, and gene assignment**

M. brandtii was used for these analyses, because numerous high quality directional RNA-seq with high coverage are publically available and the genome contains ~2000 *Helitron*-captured *NUBPL* insertions. Ilumina RNA-seq reads (200 bp, paired) from the kidney, liver, and brain tissues of *M. brandtii* (SRA061140)[14] were pooled, quality-trimmed using Trimmomatic[16], and assembled (*de-novo* and genome-guided) using Trinity (r20140413[17, 18]). The resulting assemblies from the two analyses were combined and alternative splicing analysis was performed using Program to Assemble Spliced Alignments (PASA_r20140417)[18, 19, 20]. The relative abundance of each transcript (FPKM) was determined using RNA-Seq by Expectation-Maximization (RSEM; v.1.2.12)[21]. Transcripts lacking splicing information (and thus directionality information), abundance of FPKM <0.5, and total length <200 bp were removed from the assembly, resulting in the final *M. brandtii* transcriptome assembly. Transcripts were assigned to genes by intersecting genomic coordinates with the current genome annotation (Bedtools; v.2.22.1[22]) and by verifying homology to known transcripts of that gene using BLAST. Coding potential for each transcript was determined as having a predicted ORF >100 amino acids[18]. Tissue specificity for each transcript was also determined, and a transcript was considered to be tissue specific if its FPKM value was >0.5 in only one or two of the three examined tissues.

Supplementary Note 17

Identifying *Helitron*-captured *NUBPL* promoter (NUBPL-HCP) driven transcripts in *M. brandtii*

The genomic coordinates of *Helitrons* containing the captured *NUBPL* promoter were intersected with the coordinates of the assembled transcripts. We used stringent criteria to ensure that the transcript was detectable (FPKM >0.5), that it had strand-specificity, and that the *NUBPL* promoter itself was within 1 kb upstream of the TSS[23]. Transcripts with an *NUBPL* promoter-containing *Helitron* located were classified as candidate *NUBPL-HCP* driven transcripts. Transcripts whose TSS was provided by the *NUBPL* promoter-containing *Helitron* were considered to be authenticated *NUBPL-HCP* driven transcripts. Those genes that had at least one transcript putatively driven by a *Helitron* were included in a GO Term Analysis Enrichment Analysis and terms were considered significant if their *p*-values were less than 0.05[24, 25]. Each *NUBPL* promoter within 1 kb of the TSS of a transcript with FPKM > 0.5 was analyzed for promoter motifs such as TATA, CAAT, and GC boxes, and predicted transcription factor (TF) binding sites using GPMiner[26].

Supplementary Note 18

Determining enrichment/depletion of *Helitrons* in *M. brandtii* in regions +/- 1 kb to transcription start sites (TSS)

To obtain coordinates corresponding to a 2-kb interval centered on the TSSs of *M. brandtii* genes, we extracted coordinates for -1 kb and +1 kb relative to the TSSs from our *M. brandtii* gene assemblies. We then intersected these coordinates with those of known *Helitron* insertions in *M. brandtii* (RepeatMasker, see above) using Bedtools, and determined enrichment or depletion via Fisher's Exact test ($\alpha = 0.05$)[22]. Results were considered significant if the two-tailed *p*-value was < 0.05, and the direction of the significance (enriched or depleted) was determined via the *p*-value of the appropriate one-tailed test.

Supplementary References

1. Sabouri N, McDonald KR, Webb CJ, Cristea IM, Zakian VA. DNA replication through hard-to-replicate sites, including both highly transcribed RNA Pol II and Pol III genes, requires the S. pombe Pfh1 helicase. *Genes & development* **26**, 581-593 (2012).
2. Steinacher R, Osman F, Dalgaard JZ, Lorenz A, Whitby MC. The DNA helicase Pfh1 promotes fork merging at replication termination sites to ensure genome stability. *Genes & development* **26**, 594-602 (2012).
3. Wilson MA*, et al.* Pif1 helicase and Poldelta promote recombination-coupled DNA synthesis via bubble migration. *Nature* **502**, 393-396 (2013).
4. Pritham EJ, Feschotte C. Massive amplification of rolling-circle transposons in the lineage of the bat Myotis lucifugus. *Proceedings of the National Academy of Sciences of the United States of America* **104**, 1895-1900 (2007).
5. Thomas J, Phillips CD, Baker RJ, Pritham EJ. Rolling-circle transposons catalyze genomic innovation in a Mammalian lineage. *Genome biology and evolution* **6**, 2595-2610 (2014).
6. Jurka J, Klonowski P, Dagman V, Pelton P. CENSOR--a program for identification and elimination of repetitive elements from DNA sequences. *Computers & chemistry* **20**, 119-121 (1996).
7. Liu ZJ, Moav B, Faras AJ, Guise KS, Kapuscinski AR, Hackett PB. Development of expression vectors for transgenic fish. *Bio/technology* **8**, 1268-1272 (1990).
8. Grabundzija I*, et al.* Comparative analysis of transposable element vector systems in human cells. *Mol Ther* **18**, 1200-1209 (2010).
9. Baykov AA, Evtushenko OA, Avaeva SM. A malachite green procedure for orthophosphate determination and its use in alkaline phosphatase-based enzyme immunoassay. *Anal Biochem* **171**, 266-270 (1988).
10. Ewing AD, Kazazian HH, Jr. High-throughput sequencing reveals extensive variation in human-specific L1 content in individual human genomes. *Genome research* **20**, 1262-1270 (2010).

11. Langmead B, Trapnell C, Pop M, Salzberg SL. Ultrafast and memory-efficient alignment of short DNA sequences to the human genome. *Genome biology* **10**, R25 (2009).
12. Edgar RC. MUSCLE: multiple sequence alignment with high accuracy and high throughput. *Nucleic Acids Res* **32**, 1792-1797 (2004).
13. Altschul SF, Gish W, Miller W, Myers EW, Lipman DJ. Basic local alignment search tool. *J Mol Biol* **215**, 403-410 (1990).
14. Seim I*, et al.* Genome analysis reveals insights into physiology and longevity of the Brandt's bat Myotis brandtii. *Nature communications* **4**, 2212 (2013).
15. Kapusta A*, et al.* Transposable elements are major contributors to the origin, diversification, and regulation of vertebrate long noncoding RNAs. *PLoS genetics* **9**, e1003470 (2013).
16. Lohse M*, et al.* RobiNA: a user-friendly, integrated software solution for RNA-Seq-based transcriptomics. *Nucleic Acids Res* **40**, W622-627 (2012).
17. Grabherr MG*, et al.* Full-length transcriptome assembly from RNA-Seq data without a reference genome. *Nature biotechnology* **29**, 644-652 (2011).
18. Haas BJ*, et al.* De novo transcript sequence reconstruction from RNA-seq using the Trinity platform for reference generation and analysis. *Nature protocols* **8**, 1494-1512 (2013).
19. Haas BJ*, et al.* Improving the Arabidopsis genome annotation using maximal transcript alignment assemblies. *Nucleic Acids Res* **31**, 5654-5666 (2003).
20. Campbell MA, Haas BJ, Hamilton JP, Mount SM, Buell CR. Comprehensive analysis of alternative splicing in rice and comparative analyses with Arabidopsis. *BMC genomics* **7**, 327 (2006).
21. Li B, Dewey CN. RSEM: accurate transcript quantification from RNA-Seq data with or without a reference genome. *BMC bioinformatics* **12**, 323 (2011).
22. Quinlan AR, Hall IM. BEDTools: a flexible suite of utilities for comparing genomic features. *Bioinformatics (Oxford, England)* **26**, 841-842 (2010).
23. Andersson R*, et al.* An atlas of active enhancers across human cell types and tissues. *Nature* **507**, 455-461 (2014).
24. Mi H, Muruganujan A, Thomas PD. PANTHER in 2013: modeling the evolution of gene function, and other gene attributes, in the context of phylogenetic trees. *Nucleic Acids Res* **41**, D377-386 (2013).
25. Ashburner M*, et al.* Gene ontology: tool for the unification of biology. The Gene Ontology Consortium. *Nature genetics* **25**, 25-29 (2000).
26. Lee TY, Chang WC, Hsu JB, Chang TH, Shien DM. GPMiner: an integrated system for mining combinatorial cis-regulatory elements in mammalian gene group. *BMC genomics* **13 Suppl 1**, S3 (2012).
